# Anion Chemistry: Structure, Electrochemistry and Stability of NASICON Cathodes

**DOI:** 10.1007/s40820-026-02241-5

**Published:** 2026-06-02

**Authors:** Tingting Cai, Dongxu Yu, Xueyan Zhang, Shuangshuang Zhao, Liguang Wang

**Affiliations:** 1 Institute of Zhejiang University-Quzhou, Zheda Road 99, Quzhou, 324000 People’s Republic of China; 2 School of Materials and New Energy, South China Normal University, Shanwei, 516600 People’s Republic of China; 3 College of Chemical and Biological Engineering, Zhejiang University, Hangzhou, 310058 People’s Republic of China

**Keywords:** Sodium‐ion batteries, Interfacial stability, Crystal structure, Sodium storage mechanism, Synthesis and modification strategies

## Abstract

The development of high‐energy‐density sodium‐ion batteries places stringent requirements on cathode materials to simultaneously achieve high operating voltages, rapid Na^+^ ions transport, and long‐term structural and interfacial stability. Polyanionic NASICON‐type frameworks have emerged as a compelling cathode platform due to their robust three‐dimensional Na^+^ ions diffusion networks, strong inductive effects, and excellent thermal stability. Within this materials family, fluorophosphate NASICON cathodes offer elevated redox potentials, while targeted anion chemistry modulation provides an effective strategy to tune electronic structure, ion migration, and interfacial reactivity. Representative vanadium‐based fluorophosphate cathodes, Na_3_V_2_(PO_4_)_2_F_3_ (NVPF) and Na_3_V_2_O_2_(PO_4_)_2_F (NVOPF), exhibit closely related crystal frameworks yet display distinct sodium storage behavior and degradation characteristics under high‐voltage operation. In NVOPF, partial substitution of F^−^ by O^2−^ enhances electronic conductivity and Na^+^ ions transport kinetics, while NVPF maintains a higher redox potential associated with the V^3+^/V^4+^ couple. This review critically compares NVPF‐ and NVOPF‐based cathodes in terms of crystal structure, sodium storage mechanism, synthesis and modification strategies, and high‐voltage cathode/electrolyte interfacial stability. By correlating structural chemistry with electrochemical and interfacial evolution, this work provides general insights and design guidelines for high‐voltage NASICON‐type cathodes in sodium‐ion batteries.

## Highlights

- Na_3_V_2_(PO_4_)_2_F_3_ and Na_3_V_2_O_2_(PO_4_)_2_F are systematically compared to elucidate the influence of subtle anion chemistry differences on crystal structure and Na^+^ ions diffusion pathways.
- Synthesis and modification strategies are critically evaluated to clarify the relationships among structure, properties, and performance in fluorophosphate NASICON cathodes.
- Voltage-driven interfacial degradation mechanisms are analyzed, providing transferable insights for designing durable high-voltage sodium-ion battery cathodes.

## Graphical abstract

**Figure:**
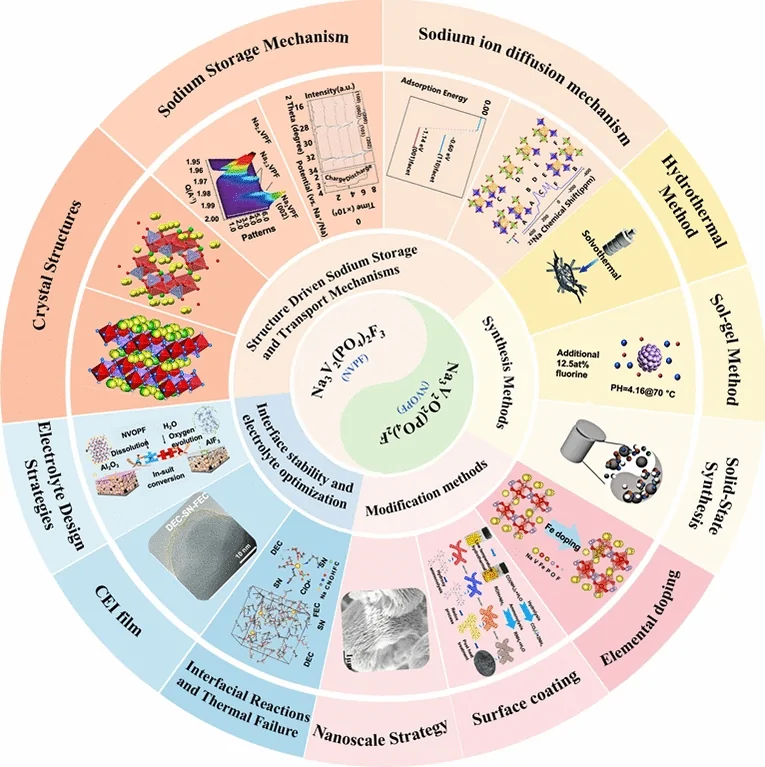

## Introduction

With the increasing demand for large-scale energy storage driven by renewable energy integration and grid stabilization, sodium-ion batteries (SIBs) have emerged as a promising alternative to lithium-ion batteries owing to the natural abundance, low cost, and wide geographical distribution of sodium resources [1–4]. However, the relatively large ionic radius of Na^+^ ions and sluggish reaction kinetics pose significant challenges to the development of high-performance cathode materials with high energy density, long cycle life, and robust safety characteristics [5–7]. Therefore, the rational design of cathode materials that concurrently enable fast Na^+^ ions transport, high operating voltage, and robust structural stability remains a central pursuit in SIB research.

Among the various cathode material families explored for SIBs, phosphate-based compounds have attracted extensive attention due to their excellent thermal stability, strong covalent bonding, and robust structural frameworks [8–11]. In particular, NASICON-type materials are regarded as one of the most promising cathode systems owing to their open three-dimensional frameworks composed of corner-sharing MO_6_ octahedra and PO_4_ tetrahedra. This unique structural motif provides continuous Na^+^ ions diffusion pathways, accommodates repeated ion insertion/extraction with minimal lattice strain, and ensures good structural reversibility during long-term cycling [12–14]. As a result, NASICON-type cathodes exhibit superior safety characteristics and structural durability compared with layered oxides and Prussian blue analogues, especially under high-rate and high-temperature conditions.

Within the NASICON family, fluorophosphate-based vanadium cathodes, represented by Na_3_V_2_(PO_4_)_2_F_3_ (NVPF) and Na_3_V_2_O_2_(PO_4_)_2_F (NVOPF), have recently attracted considerable interest due to the high operating voltages and favorable electrochemical stability. Compared with conventional NASICON cathodes (Na_3_V_2_(PO_4_)_3_ (NVP)), which operates at ~ 3.4 V, the incorporation of highly electronegative F^−^ into NVPF elevates the redox potential of the V^3+^/V^4+^ couple to ~ 4.1 V via a strong inductive effect, leading to a significantly higher energy density [15–17]. Furthermore, the partial substitution of F^−^ with O^2−^ in NVOPF creates a mixed-anion framework that not only maintains a high operating voltage (~ 3.8 V) but also enhances electronic conductivity and Na^+^ transport kinetics, outperforming many phosphate-based counterparts in rate capability. While other NASICON variants, such as iron or manganese-based phosphates, offer advantages in terms of cost and elemental abundance, the fluorophosphates NVPF and NVOPF stand out for their unique combination of high operating voltage, robust structural stability, and tunable electrochemical kinetics, making them ideal model systems for studying the interplay between anion chemistry and high-voltage performance.

To further optimize the electrochemical performance of fluorophosphate NASICON cathodes, NVOPF has been developed through partial substitution of F^−^ with O^2−^ in the vanadium coordination environment [18]. This anion substitution strategy effectively modulates the electronic structure and local bonding characteristics, resulting in enhanced electronic conductivity and accelerated Na^+^ ions transport kinetics while preserving the structural integrity of the NASICON framework. As a consequence, NVOPF exhibits improved rate capability and higher accessible capacity, making it an attractive candidate for achieving high specific capacity at relatively high operating voltages [19]. Despite the structural similarity, NVPF and NVOPF display distinct electrochemical behaviors, sodium storage mechanisms, ion diffusion characteristics, and cycling stability, highlighting the critical role of anion chemistry in regulating the structure and property relationships of fluorophosphate NASICON cathodes.

Importantly, the differences between NVPF and NVOPF extend beyond bulk electrochemical performance and are strongly manifested in their interfacial reactivity and degradation pathways under high-voltage operation. The high operating voltage of NVPF, while beneficial for achieving high energy density, often exceeds the thermodynamic stability window of conventional carbonate-based electrolytes, leading to severe oxidative decomposition at the cathode/electrolyte interface [20]. This process results in the formation of cathode electrolyte interphase (CEI) layers with high impedance, continuous electrolyte consumption, and accelerated performance decay. In contrast, the slightly lower operating voltage of NVOPF reduces the thermodynamic driving force for electrolyte oxidation; however, its distinct anion chemistry may induce alternative degradation pathways, such as transition-metal dissolution and structural instability triggered by interfacial side reactions. Thus, to elucidate the long-term cycling behavior of NVPF and NVOPF cathodes, it is essential to understand the evolution of interfacial reactions at the cathode/electrolyte interface and the associated CEI chemistry.

**Fig. 1:**
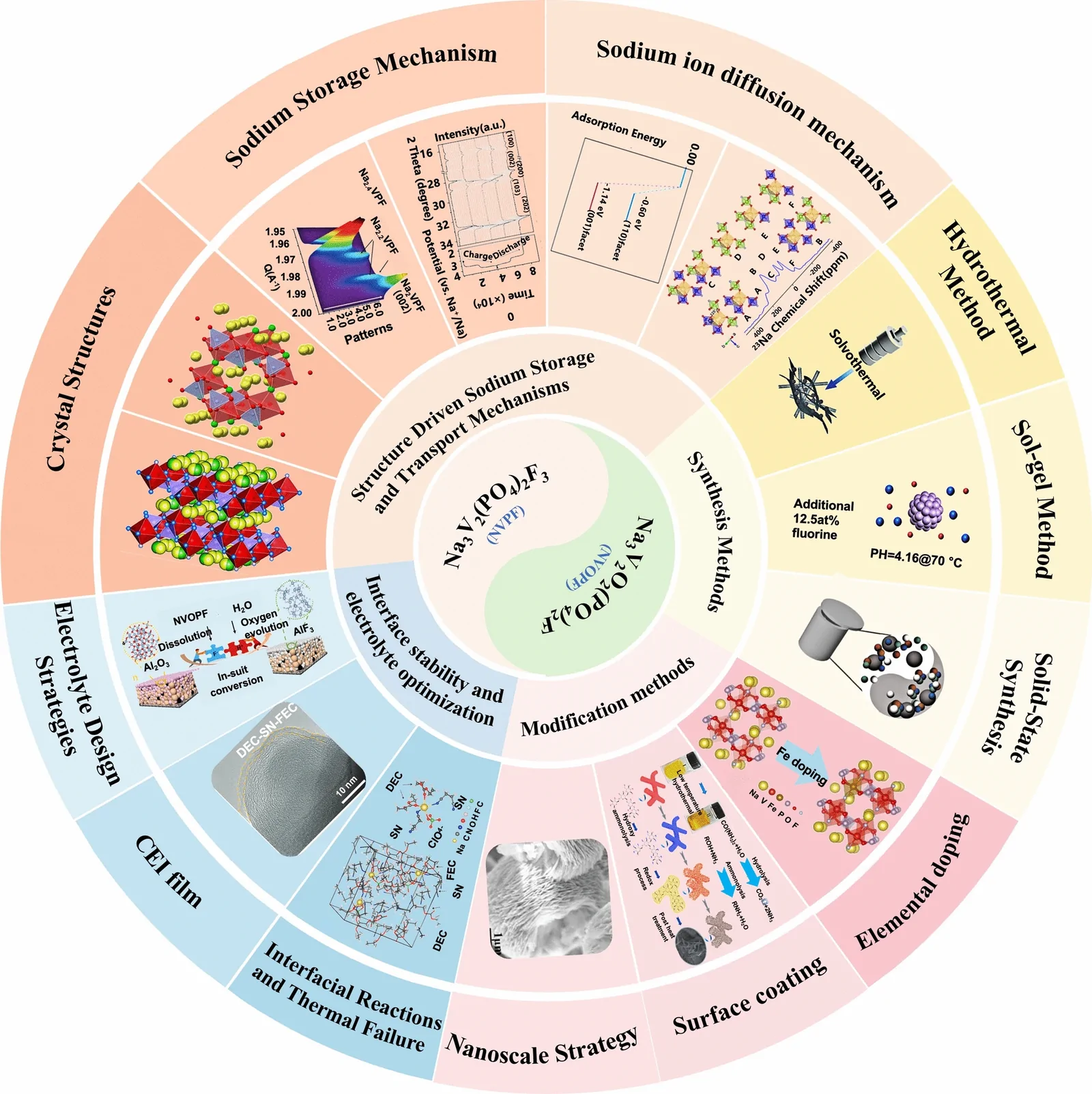
Summary and comparative study of NVPF and NVOPF cathode materials in sodium-ion batteries

Although extensive studies have been devoted to optimizing the synthesis methods, structural modifications, and electrochemical performance of NVPF- and NVOPF-based cathodes, systematic and direct comparisons between these two materials remain relatively scarce. Moreover, key scientific issues, such as the structure and property relationships governing sodium storage and diffusion, the role of anion substitution in regulating electronic structure, and the evolution of cathode/electrolyte interfacial reactions at high voltages have not yet been comprehensively summarized. A clear and unified understanding of these aspects is essential for guiding the rational design of next-generation NASICON-type cathodes with both high energy density and long-term cycling stability. In this review, a comprehensive and critical comparison of NVPF and NVOPF cathode materials was provided from the following key perspectives (Fig. 1): crystal structure characteristics, sodium storage mechanisms, synthesis strategies, modification approaches, and high-voltage cathode/electrolyte interfacial stability. By elucidating the relationships among structure, properties, and interfacial behavior, and by highlighting both the advantages and limitations, this work aims to provide mechanistic insights and practical guidance for the development of high-performance cathode materials for advanced sodium-ion batteries.

## Structure Driven Sodium Storage and Transport Mechanisms

### Crystal Structures of NVPF and NVOPF

**Fig. 2:**
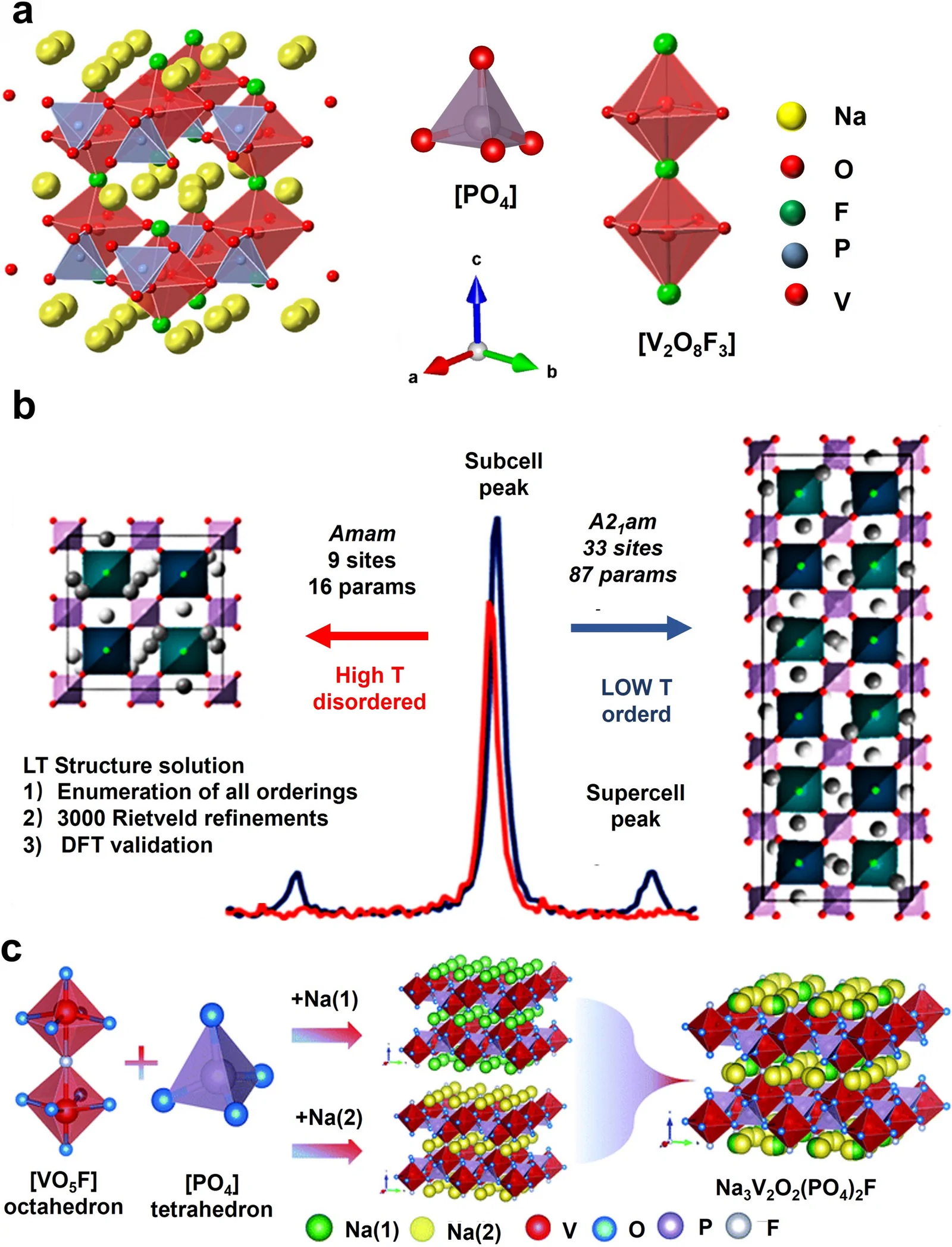
Schematic diagram of crystal structure. **a** Crystal structure of NVPF. [25]. Copyright 2022, Energy & Environmental Materials. **b** Schematic illustrating the structure solution process [22]. Copyright 2020, American Chemical Society. **c** The crystalline structure of the NVOPF. Reproduced with permission [26]. Copyright 2020, Wiley–VCH

NVPF was first reported in 1999 as a NASICON-structured material analogous to NVP with the *P4*_*2*_*/mnm* space group through a hydrothermal method. The crystal structures were determined by X-ray diffraction, with the lattice parameters of *a* = 9.047 Å, *c* = 10.705 Å, and *V* = 876.2 Å^3^. The crystal structure is built from [V_2_O_8_F_3_] dioctahedral and [PO_4_] tetrahedra, which share oxygen atoms within the ab-plane (Fig. 2a). The dihedral units are connected by F atoms, while the [PO_4_] units are connected by O atoms. Bianchini et al*.* revealed a slight orthorhombic distortion using high-resolution synchrotron diffraction [21], which was refined in the Amam space group with lattice parameters *a* = 9.02847(3) Å, *b* = 9.04444(3) Å, and *c* = 10.74666(6) Å. The crystal structure of NVPF exhibits clear temperature dependence, transitioning from a fully disordered tetragonal structure at high temperatures to a partially ordered arrangement (Fig. 2b). The NVPF adopts different polymorphs at low and high temperatures (Fig. 2c) [22]. As the temperature decreases, sodium-ion transition from a dynamically disordered state to a statically ordered configuration, which serves as the primary driving force for the symmetry-lowering phase transition of the crystal structure. Although NVPF and NVOPF both belong to the tetragonal crystal system, the crystal structures are fundamentally different. NVOPF was first synthesized via a hydrothermal method, and the crystal structure was determined by single-crystal X-ray diffraction [23]. NVOPF crystallizes in the tetragonal *I4/mmm* space group with lattice parameters of *a* = *b* = 6.3856(2) Å and *c* = 10.6119(9) Å [24].

Unlike NVPF, NVOPF is composed of [VO_5_F] octahedra and [PO_4_] tetrahedra that are alternately connected through shared oxygen atoms within the ab-plane, while fluorine atoms bridge adjacent octahedra along the c-axis to form a three-dimensional framework. Although these two polymorphs exhibit similar overall framework structures, the *P4*_*2*_*/mnm* phase displays a more ordered sodium arrangement than the *I4/mmm* phase, resulting in distinct electrochemical behavior during Na^+^ ions insertion and extraction. The pronounced structural differences between NVPF and NVOPF, particularly in space groups, vanadium coordination environments, and atomic connectivity, give rise to fundamentally different sodium storage mechanisms and ion transport behavior. Consequently, extensive efforts have been devoted to developing synthesis and regulation strategies, as rational control over crystal structure characteristics and defect chemistry plays a decisive role in tuning ionic conductivity, structural stability, and electrochemical performance.

In NVPF, the fluorine coordination environment of V^3+^ achieves a high potential of approximately 4.1 V through strong induction effects. However, the ordered arrangement of Na^+^ ions induce multiple phase transitions and limits diffusion kinetics. In NVOPF, O^2−^ substitution slightly lowers the operating potential (~ 3.8 V) while enhancing intrinsic electronic conductivity via π-electron delocalization of the V=O bond. Concurrently, Na^+^ ions site disorder suppresses intermediate-phase transitions, transforming the sodium storage mechanism from a multiphase reaction (NVPF) to a solid-solution behavior, thereby reducing diffusion barriers and improving rate capability.

### Sodium Storage Mechanism

Investigating sodium storage mechanisms provides fundamental insight into the relationship between material structure and electrochemical performance, thereby guiding rational design strategies to overcome limitations and enhance both energy density and cycling stability in sodium-ion batteries. In NVPF, sodium storage originates from its unique NASICON-type three-dimensional framework [14, 27], which features two major diffusion tunnels: an a-axis tunnel accommodating two Na^+^ ions at Na1 sites and a c-axis tunnel hosting one Na^+^ ions at the Na2 site [28]. These interconnected tunnels enable multidimensional Na^+^ ions diffusion pathways. Among the three Na^+^ ions in NVPF, only two are electrochemically active, corresponding to the reversible V^3+^/V^4+^ redox couple, as described by the ideal reaction: Na_3_V_2_(PO_4_)_2_F_3_ ↔ NaV_2_(PO_4_)_2_F_3_ + 2Na^+^ + 2e^−^. However, NVPF cathodes typically exhibit three discharge voltage plateaus at approximately 4.1, 3.6, and 3.3 V. The emergence of additional low-voltage plateaus lowers the average discharge voltage and, consequently, reduces the energy density of both the cathode material and the full cell. Therefore, suppressing or eliminating these low-voltage plateaus is crucial for maximizing the electrochemical performance of NVPF-based batteries. Effective strategies to address this issue include: (i) precisely controlling the synthesis conditions to suppress the formation of NaVPO_4_F or Na_3_V_2_(PO_4_)_3_ impurity phases that contribute to low-voltage redox activity [29]; (ii) employing elemental doping (e.g., Li^+^, Mg^2+^) to modulate the local coordination environment and Na^+^ ions/vacancy ordering, thereby reducing the population of Na^+^ ions occupying low-voltage sites [30]. These approaches collectively aim to eliminate the parasitic low-voltage reactions, thereby increasing the average operating voltage and overall energy density of NVPF-based cathodes.

**Fig. 3:**
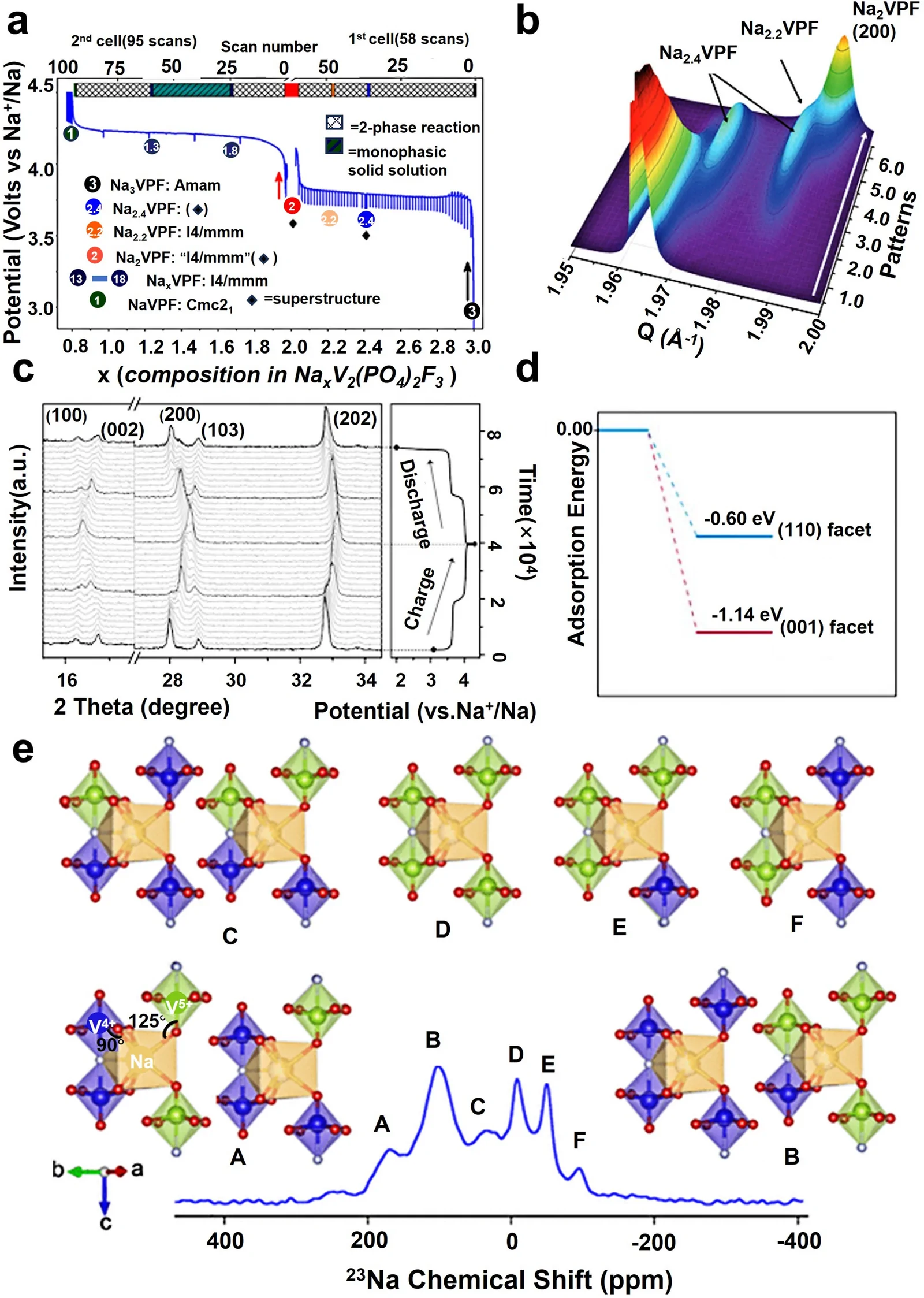
**a** Potential-composition electrochemical curves from NVPF [32]. Copyright 2015, American Chemical Society. **b** Corresponding 3D plot between the compositions NaVPF and Na_2_VPF [31]. Copyright 2019, Wiley–VCH. **c** The XRD patterns of NVPF-NTP electrode [33]. Copyright 2017, Wiley–VCH. **d** Binding energies of the two sites [34]. Copyright 2024, Royal Society of Chemistry. **e** Na coordination configurations in Na_2_VOPF [36]. Copyright 2024, Royal Society of Chemistry

In NVPF, Na^+^ ions deintercalation does not proceed via a simple single-phase solid-solution mechanism but instead involves a combination of multiphase reactions and solid-solution behavior. As shown in Fig. 3a, multiple voltage plateaus and sloping regions are observed in the charge–discharge profiles during Na^+^ ions extraction from Na_3_V_2_(PO_4_)_2_F_3_ to Na_1_V_2_(PO_4_)_2_F_3_ [31, 32]. The voltage plateaus correspond to two-phase reactions occurring at specific compositions (e.g., Na_2.4_VPF, Na_2.2_VPF, and Na_2_VPF), where the crystal structure undergoes phase transitions to form thermodynamically stable intermediate phases. These transitions are typically accompanied by Na^+^ ions and/or charge ordering, resulting in regular occupation of specific crystallographic sites (Fig. 3b). In contrast, the sloping voltage regions are indicative of single-phase solid-solution reactions, in which Na^+^ ions are continuously deintercalated/intercalated within the host framework, leading to gradual changes in lattice parameters while preserving the overall space group symmetry.

Unlike NVPF, Na^+^ ions intercalation/deintercalation in NVOPF is predominantly governed by a solid-solution mechanism. During electrochemical cycling, the lattice structure evolves continuously without obvious phase separation. NVOPF contains two crystallographically distinct Na sites: the Na1 site coordinated by five oxygen atoms and one fluorine atom, and the Na2 site coordinated by six oxygen atoms. Both sites are partially occupied by disordered Na^+^ ions forming circular arrangements within the ab-plane. Electrochemically, NVOPF exhibits two charge–discharge plateaus at approximately 3.6 and 4.0 V [33]. The lower-voltage process corresponds to Na_3_V_2_O_2_(PO_4_)_2_F ⇌ Na_2_V_2_O_2_(PO_4_)_2_F + Na^+^ + e^−^, while the higher-voltage reaction follows Na_3_V_2_O_2_(PO_4_)_2_F ⇌ NaV_2_O_2_(PO_4_)_2_F + Na^+^ + e^−^ [33]. During charging, characteristic diffraction peaks (e.g., the (002) reflection) in in-situ XRD patterns exhibit systematic splitting and shifting (Fig. 3c), indicating a sequential two-phase transition (Na_3_ → Na_2_ → Na_1_) that is consistent with the proposed reaction mechanism.

### Sodium-Ion Diffusion Mechanism

The rate capability and power density of Na^+^ ions batteries are strongly governed by the diffusion kinetics of Na^+^ ions within the cathode material. Therefore, a comprehensive understanding of Na^+^ ions diffusion pathways and associated energy barriers is essential for the rational design and optimization of high-performance cathode materials. In NVPF, it has been elucidated that the efficient diffusion of Na^+^ ions in NVPF cathodes originates from the preferential adsorption on specific crystal planes. The adsorption energy of Na^+^ ions on the exposed (002) active crystal plane (− 1.14 eV) is significantly lower than that on the (110) plane (− 0.60 eV) (Fig. 3d), indicating a stronger tendency for stable Na^+^ ions adsorption on the (002) plane, which serves as a favorable starting point for subsequent inward migration [34]. Subsequently, the migration process from the crystal surface into the bulk also differs substantially. The diffusion energy barrier for Na^+^ ions entering via the (002) plane is only 0.43 eV, markedly lower than the 0.66 eV barrier associated with the (110) plane. This suggests that the (002) plane acts as a kinetically favorable entry point for Na^+^ intercalation. Similarly, Song et al*.* identified a dominant Na^+^ ions transport pathway located between the (002) planes stacked perpendicular to the *c*-axis [35]. This continuous and well-defined channel enables rapid, low-resistance two-dimensional Na^+^ ions diffusion. Therefore, enhancing the exposure of the (002) crystal plane represents an effective strategy to optimize Na^+^ ions transport. Unlike NVPF, Na^+^ ions migration in NVOPF predominantly occurs within the ab-plane through a vacancy-assisted mechanism. This pathway features an exceptionally low diffusion energy barrier (0.15–0.31 eV), effectively functioning as an “ion highway” [36]. In contrast, diagonal or interlayer migration is energetically unfavorable, with activation barriers exceeding 1.7 eV. In addition, six distinct Na^+^ ions chemical environments were identified in the intermediate charged state, analogous to six available “seats” within the crystal framework [36]. These environments are governed by the local valence states of neighboring vanadium ions (V^4+^ or V^5+^). V^5+^-rich regions exert stronger electrostatic attraction, resulting in more tightly bound Na^+^ ions, whereas environments with fewer surrounding V^5+^ ions provide weaker binding. Consequently, Na^+^ ions occupying sites with lower V^5+^ ions coordination (e.g., sites A, B, and C) exhibit faster migration kinetics, with activation energies as low as 0.1–0.2 eV (Fig. 3e). Together, these findings provide atomic-level insight into Na^+^ ions transport in NVOPF and explain its favorable electrochemical kinetics.

Overall, the Na^+^ ions diffusion mechanisms in NVPF and NVOPF are structurally distinct. NVPF relies on anisotropic diffusion mediated by specific exposed crystal planes, where the (002) plane serves as a kinetically favorable entry site with a relatively low migration barrier. In contrast, NVOPF benefits from intrinsic two-dimensional diffusion pathways within the ab-plane, enabled by its tetragonal symmetry and disordered Na^+^ ions/vacancy distribution, resulting in lower activation energies. The key factors governing these differences include the space group symmetry, the local coordination environment of vanadium (V–O vs. V–F), and the degree of Na^+^ ions/vacancy ordering, all of which are dictated by the underlying anion chemistry. These fundamental insights into the distinct diffusion mechanisms provide a theoretical foundation for targeted optimization strategies, such as crystal plane engineering in NVPF and maintaining the structural integrity of the 2D diffusion planes in NVOPF, which are typically realized through synthesis and modification approaches.

## Synthesis Methods

The excellent specific capacity of NVPF and NVOPF depends on factors such as material morphology and defect characteristics, prompting investigations into their synthesis methods. Researchers have changed the morphology and structure of two materials by adopting different synthesis methods, such as the solvothermal method [37, 38], sol–gel method [37], and ball milling method [39, 40], with the purpose of obtaining structurally stable sodium-ion battery cathodes with excellent electrochemical performance.

### Hydrothermal Method/Solvothermal Method

Hydrothermal strategies, including hydrothermal and solvothermal methods, are widely employed for the synthesis of NVPF and NVOPF materials. In these processes, vanadium, sodium, phosphorus, and fluorine precursors are dissolved in water or appropriate solvents together with citric acid in defined stoichiometric ratios, followed by heating and stirring to form a homogeneous solution. The solution is then transferred to a sealed autoclave and reacted at controlled temperatures for specific durations. After the reaction, the products are washed and calcined to obtain phase-pure materials [41, 42]. By tuning key parameters such as temperature, reaction time, pH, and precursor concentration, the crystallinity, particle size, and morphology can be effectively regulated [38, 43–45]. As a result, NVPF and NVOPF synthesized via hydrothermal routes typically exhibit high discharge capacities and favorable rate performance. This section further discusses how hydrothermal conditions influence the structural and morphological evolution of NVPF and NVOPF materials.

In hydrothermal synthesis, temperature is a critical parameter for precisely controlling particle size, morphology, and phase formation. NVOPF is typically synthesized at relatively mild temperatures ranging from 120 to 180 °C, whereas NVPF generally requires higher temperatures of 130 to 200 °C. This difference arises because the formation of NVPF necessitates higher thermal energy to reduce vanadium precursors to V^3+^ and to drive the reaction toward the thermodynamically stable, highly crystalline NASICON structure, in contrast to the metastable, mixed-valent NVOPF phase. Insufficient reaction temperatures fail to provide adequate energy for the ordered arrangement of V, PO_4_^3−^, O^2−^, and F^−^ ions, often resulting in incomplete reactions and the formation of impurity phases. Conversely, excessively high temperatures can induce fluorine loss, leading to lattice distortion and the emergence of secondary phases.

**Fig. 4:**
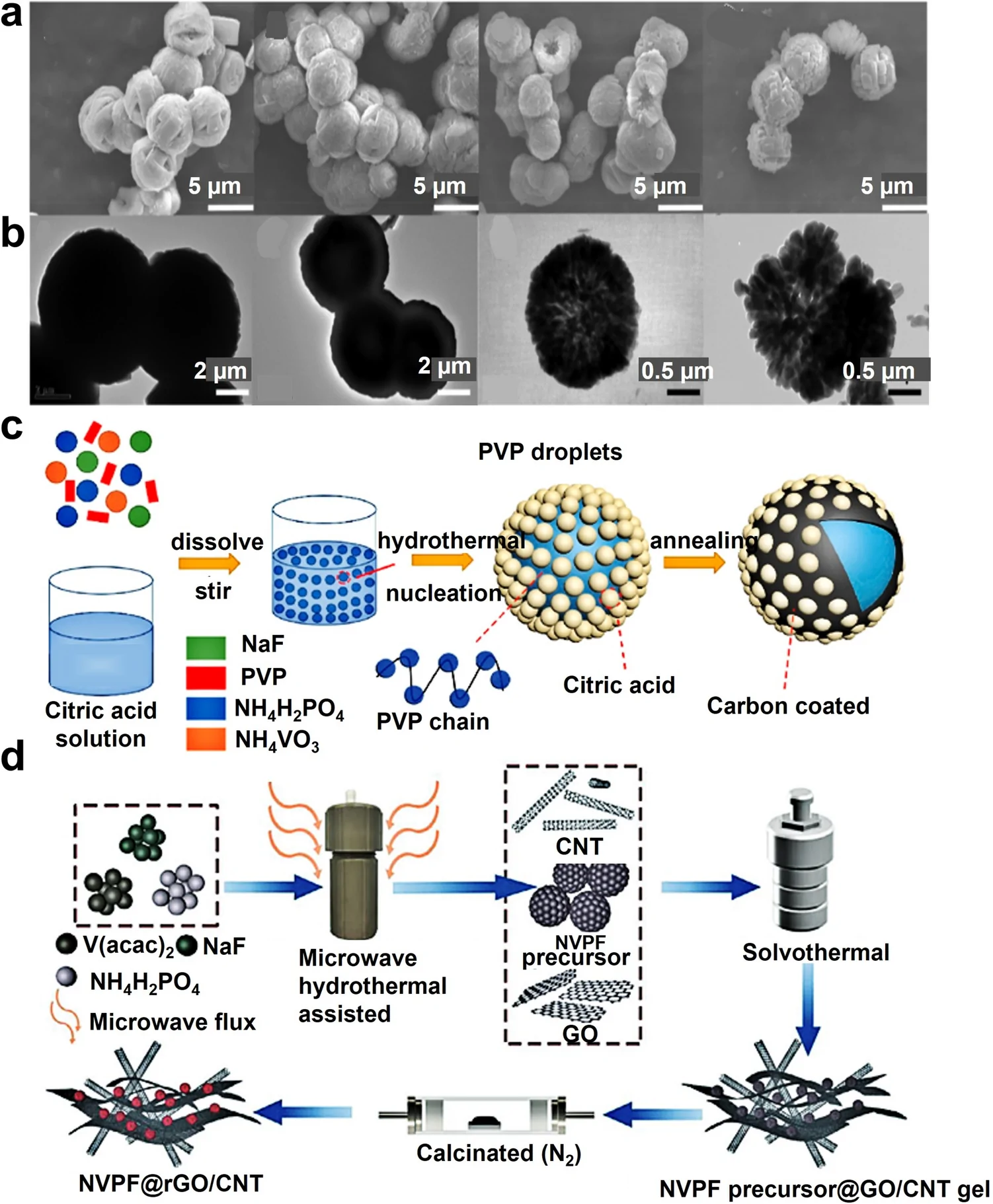
**a, b** Typical SEM and TEM images of NVPF [46]. Copyright 2019, American Chemical Society. **c** Schematic illustration for the synthetic procedure of NVPF@rGO/CNT [48]. **d** Schematic diagram of NVMPF@C microsphere [49]. Copyright 2021, Elsevier

To elucidate the effect of temperature on NVOPF morphology, Shen et al. synthesized NVOPF at 80, 120, 180, and 240 °C. When the temperature exceeded 240 °C, structural collapse or excessive particle growth occurred, leading to deteriorated electrochemical performance [46]. SEM and TEM analyses (Fig. 4a, b) revealed a temperature-induced morphological evolution from solid particles to hollow structures between 180 and 240 °C. This transformation demonstrates that hydrothermal temperature serves as a critical parameter for tailoring the internal architecture of NVOPF. The resulting loose internal packing promotes hollow structure formation through an Ostwald ripening process. A similar mechanism has been exploited for NVOPF morphology control. Zhao et al*.* fabricated hierarchical multi-hollow NVOPF nanospheres via a template-free solvothermal route, further confirming the role of reaction temperature in directing hollow structure formation. Benefiting from the synergistic effects of hollow nanostructures and hierarchical porosity, the resulting NVOPF-hs exhibited ultrafast kinetics and excellent cycling stability [47].

In comparison, NVPF can form three-dimensional architectures even at relatively low hydrothermal temperatures. NVPF@rGO/CNT composites with a three-dimensional conductive network were synthesized via microwave-assisted hydrothermal treatment at 130 °C (Fig. 4c) [48]. Under these conditions, uniform nucleation and controlled growth of NVPF nanoparticles with sizes of approximately 300 nm were achieved, effectively avoiding incomplete crystallization at lower temperatures as well as particle agglomeration or excessive grain growth at elevated temperatures. The resulting optimized morphology provides a favorable structural basis for the construction of an interconnected conductive network. At a higher hydrothermal temperature of 200 °C, porous NVPF microspheres were obtained using polyvinylpyrrolidone as a soft template (Fig. 4d) [49]. The increased thermal energy facilitates complete precursor reactions, nanoparticle dissolution–reprecipitation, and template-directed self-assembly, leading to the formation of porous microspheres with uniform size, smooth surfaces, and abundant internal pores. Such a hierarchical porous morphology increases the specific surface area and promotes Na^+^ ions diffusion, thereby enhancing electrochemical performance. In summary, hydrothermal temperature plays a decisive role in governing the morphology and microstructural evolution of NVPF materials. Systematic investigation of temperature-dependent effects is therefore one of the most fundamental and critical steps in tailoring targeted morphologies and optimizing the hydrothermal synthesis of high-performance sodium-ion battery cathodes.

**Fig. 5:**
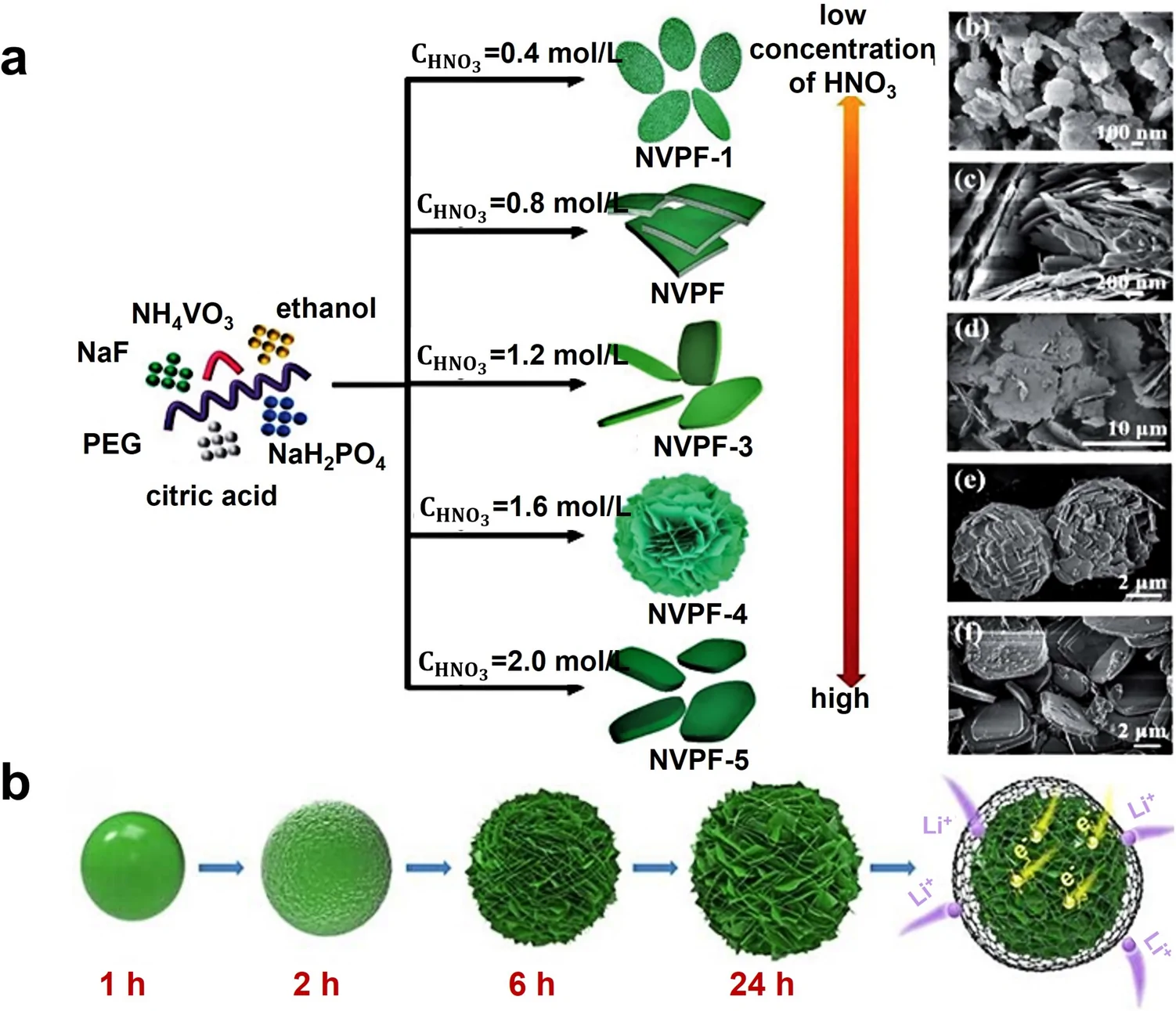
**a** Schematic illustration for the synthesis of NVPF@Rx [50]. Copyright 2022, Royal Society of Chemistry. **b** Illustration of the formation process of NVOPF micro-flowers and NVOPF/G [52]. Copyright 2021, Wiley

In addition to temperature, the concentration of the hydrothermal solution plays a crucial role in determining material morphology. The influence of acid concentration on morphology evolution was systematically investigated by introducing HNO_3_ with concentrations ranging from 0.4 to 2.0 mol L^−1^ during the synthesis of NVPF@Rx (Fig. 5a) [50]. At a relatively low HNO_3_ concentration of 0.4 mol L^−1^, NVPF-1 exhibits a leaf-like morphology composed of nanoparticles with sizes of approximately 300 nm. As the acid concentration increases, the nanosheets gradually grow in lateral dimensions and begin to stack in a two-dimensional manner. When the HNO_3_ concentration reaches 1.2–2.0 mol L^−1^, the morphology further evolves from two-dimensional nanosheets into flower-like architectures formed via nanosheet self-assembly. At even higher acid concentrations, intensified particle agglomeration and densification dominate the growth process, ultimately resulting in the formation of dense micro-block structures.

Solution concentration strongly influences morphology by regulating nucleation and growth kinetics [51]. At low concentrations, weak driving forces and limited nucleation sites favor slow, anisotropic crystal growth, resulting in low-dimensional structures such as nanowires, nanorods, or nanosheets. In contrast, high concentrations induce rapid nucleation and the formation of numerous crystal nuclei, promoting the development of three-dimensional architectures. Therefore, precise control over solution concentration enables directional tuning of material morphology from low- to high-dimensional structures. In addition to temperature and concentration, reaction time is another critical parameter governing structural evolution. For example, during the concentrated solvothermal synthesis of NVOPF using propylene glycol as the solvent, a gradual increase in microsphere diameter and nanosheet length was observed with prolonged reaction time [52]. The well-defined NVOPF micro-flowers composed of assembled nanosheets were obtained (Fig. 5b) after 24 h. This evolution can be attributed to the Ostwald ripening mechanism, in which smaller particles dissolve and redeposit onto larger ones, yielding thermodynamically more stable hierarchical structures.

Overall, temperature and solution concentration are key parameters controlling the morphology and, consequently, the electrochemical performance of NVPF and NVOPF materials. Table 1 summarizes representative synthesis conditions for hydrothermally prepared NVPF- and NVOPF-based materials. Although the hydrothermal method is cost-effective and facile, its reliance on high-pressure conditions poses safety concerns and limits large-scale production. Further optimization and technical refinement of hydrothermal synthesis routes are therefore required to enhance their practical applicability.

**Table 1:** Summary of temperature, solvent, and property effects in hydrothermal and solvothermal synthesis of NVPF and NVOPF

Materials	Synthesis method	Solvents	Temperature (℃)	Electrochemical performance	References
NVPF@RuO_2_	Solvothermal	HNO_3_	180	20 C 115.8 mAh g^−1^ 100%	[50]
HM-NVPF@CN	Solvothermal	EG	170	10 C 70 mAh g^−1^ 80%	[53]
c-NVPF@NC	Hydrothermal	H_2_O	170	10 C 73.6 mAh g^−1^	[54]
0.1Li-NVPF/CNTs-Al (C)	Hydrothermal	H_2_O	130	30 C 30000 cycles 64.1%	[30]
NVPF@rGO	Hydrothermal	H_2_O	170	50 C 73.7 mAh g^−1^	[55]
NVPF-HMS	Solvothermal	EG	200	0.1 C 119 mAh g^−1^	[56]
NVPF@C-10PV	Hydrothermal	H_2_O	200	1 C 106.1 mAh g^−1^ 83%	[49]
NVPF	Solvothermal	EG:H_2_O = 2:1	200	5 C 67.4 mA h g^−1^	[57]
NVPF@rGO	Hydrothermal	H_2_O	130	0.5 C 126.9 mAh g^−1^	[58]
NVPF@C@rGO	Hydrothermal	H_2_O	170	100 C 64 mAh g^−1^	[59]
NVPF@3Dc	Hydrothermal	H_2_O	170	0.2 C 131.5 mAh g^−1^ 86%	[60]
NVPOF@G	Hydrothermal	H_2_O	180	50 C 82.2 mAh g ^−1^ 87.7%	[61]
tNVPF@C	Solvothermal	DMF	180	5 C 300 cycles ~ 80%	[62]
NVPF@CNTs	Hydrothermal	DMF	180	0.2 C 117.6 mA h g^−1^	[63]
NVPF/rGO	Hydrothermal	H_2_O	180	0.1 C 127 mA h g^−1^ 99%	[64]
PCNF@NVOPF NR	Solvothermal	DMF	180	1 A 1200 cycles 94%	[65]
NVOPF	Solvothermal	H_2_O + EG	180	660 Wh kg^−1^	[24]
NVOPF@rGO	Solvothermal	DMF	180	50 mA g^−1^ 50 cycles 66.7%	[36]
NVOPF	Solvothermal	1,2-Propanediol	180	5 C 1000 cycles 84.8 mAh g^−1^	[52]
Cr/Mn-NVOPF	Solvothermal	EG	180	20 C 87 mAh g^−1^	[66]
NVN_0.5_POF/rGO	Solvothermal	DMF	180	10 C 72 mAh g^−1^ 65.2%	[67]

### Sol–Gel Method

**Fig. 6:**
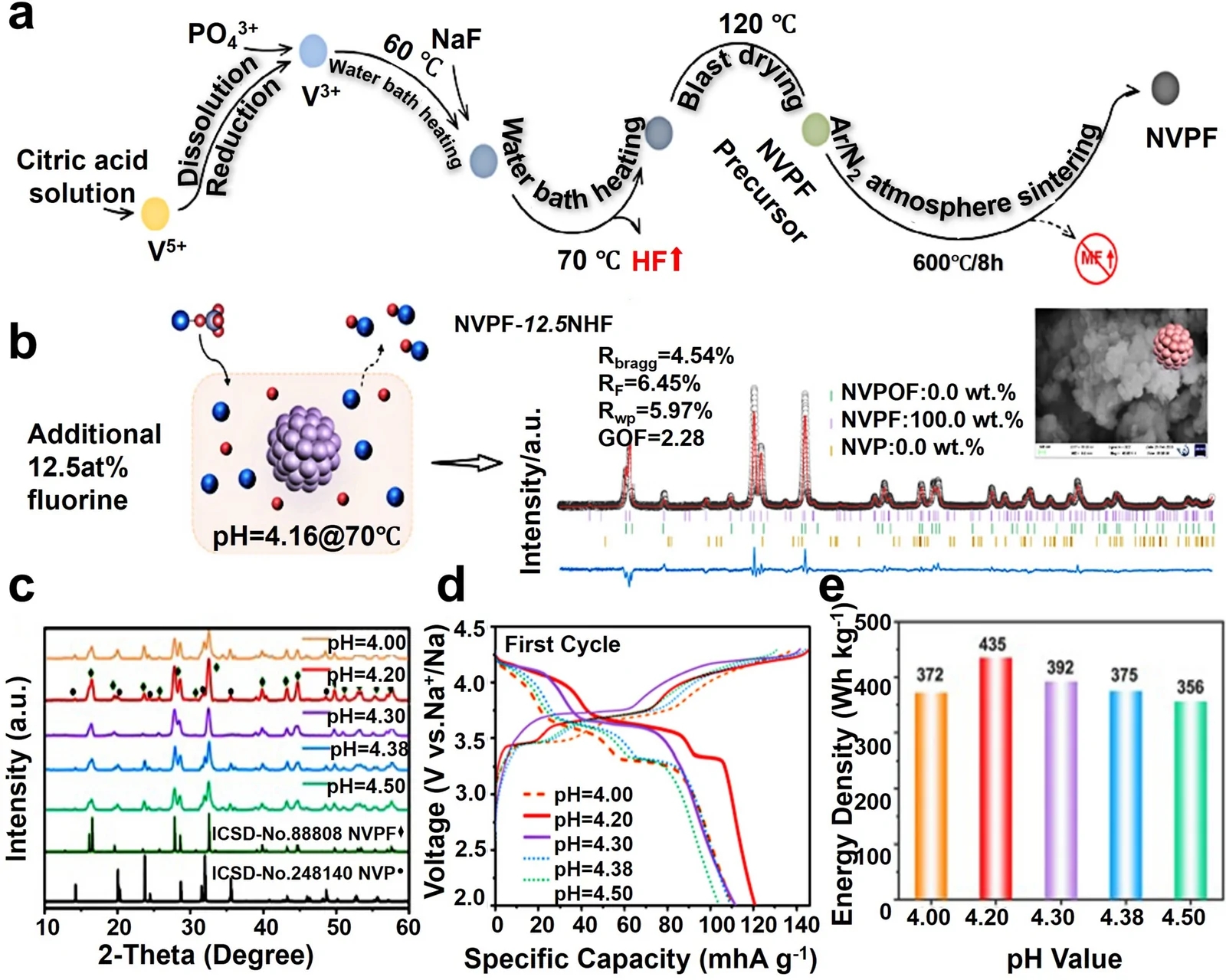
**a** Schematic diagram of the fluorine loss path [69]. Copyright 2025, Wiley. **b** Schematic diagram of the dynamic fluorine supplementation strategy [69]. Copyright 2025, Wiley. **c** XRD patterns of NVPF@rGO samples [70]. Copyright 2023, Elsevier. **d** The initial galvanostatic charge/discharge profiles [70]. Copyright 2023, Elsevier. **e** Corresponding specific energy density at 1C of the NVPF@rGO samples [70]. Copyright 2023, Elsevier

The sol–gel method is also widely used for the synthesis of NVPF, as it enables the preparation of uniform nanoparticles with sizes of approximately 200 nm and offers good process controllability. In this method, the pH of the sol is a critical parameter that strongly affects system stability and the composition of the final product [68]. This sensitivity arises because fluoride ions (F^−^) readily react with protons (H^+^) to form volatile HF, leading to fluorine loss. To mitigate this issue, two primary strategies are commonly adopted: regulating the pH to suppress HF formation, for example, by introducing ammonia to reduce H^+^ ions concentration. Consequently, pH control plays a decisive role in determining phase purity and electrochemical performance by governing fluorine retention in NVPF. Wang et al. proposed a dynamic fluorine-compensation strategy using NH_4_F as a dual-functional additive during the sol–gel drying process [69]. Under strongly acidic conditions, excessive fluorine volatilization leads to impurity phases that deteriorate operating voltage and capacity (Fig. 6a). By stabilizing the pH at an optimized value of 4.16, fluorine loss was effectively suppressed, yielding a high-purity NVPF phase (Fig. 6b) with markedly improved specific capacity, energy density, high-rate capability, and long-term cycling stability.

Beyond fluorine retention, pH regulation also influences crystal growth behavior and impurity suppression. Adjusting the precursor pH to approximately 4.20 modulates selective H^+^ adsorption on different crystal planes, thereby altering surface energies and growth rates of both the NVPF main phase and the NVP impurity phase [70]. This effect effectively suppresses NVP formation and promotes the growth of highly crystalline NVPF (Fig. 6c), eliminating the unfavorable ~ 3.4 V low-voltage plateau associated with NVP impurities (Fig. 6d). As a result, the optimized material exhibits a higher average operating voltage, enhanced energy density of up to 435 Wh kg^−1^ (Fig. 6e), and improved cycling stability. These findings collectively highlight pH regulation as an effective and versatile strategy for achieving high-performance NVPF cathode materials. In contrast, the sol–gel method is rarely applied to the synthesis of NVOPF, primarily due to differences in fluorine stability and precursor chemistry. While NVPF exhibits relatively robust structural tolerance to synthesis conditions, the presence of V–O bonds in NVOPF renders its precursors highly sensitive to the liquid-phase environment of the sol–gel process. Moreover, fluoride ions are prone to volatilization during thermal treatment, leading to compositional deviations and reduced phase purity. The hydrothermal and solvothermal methods provide a closed reaction environment that more effectively controls fluorine incorporation, making them more suitable for the reliable synthesis of NVOPF than the more complex and less controllable sol–gel route.

### Solid-State Synthesis

Compared to hydrothermal, solvothermal, and sol–gel methods, solid-state synthesis is a solvent-free approach. This method eliminates issues associated with solvent/liquid waste contamination, thereby reducing environmental impact and preventing solvent-derived impurities in the final product [71]. However, challenges such as uncontrollable morphology, particle inhomogeneity, or coating defects remain significant technical barriers for this technique.

**Fig. 7:**
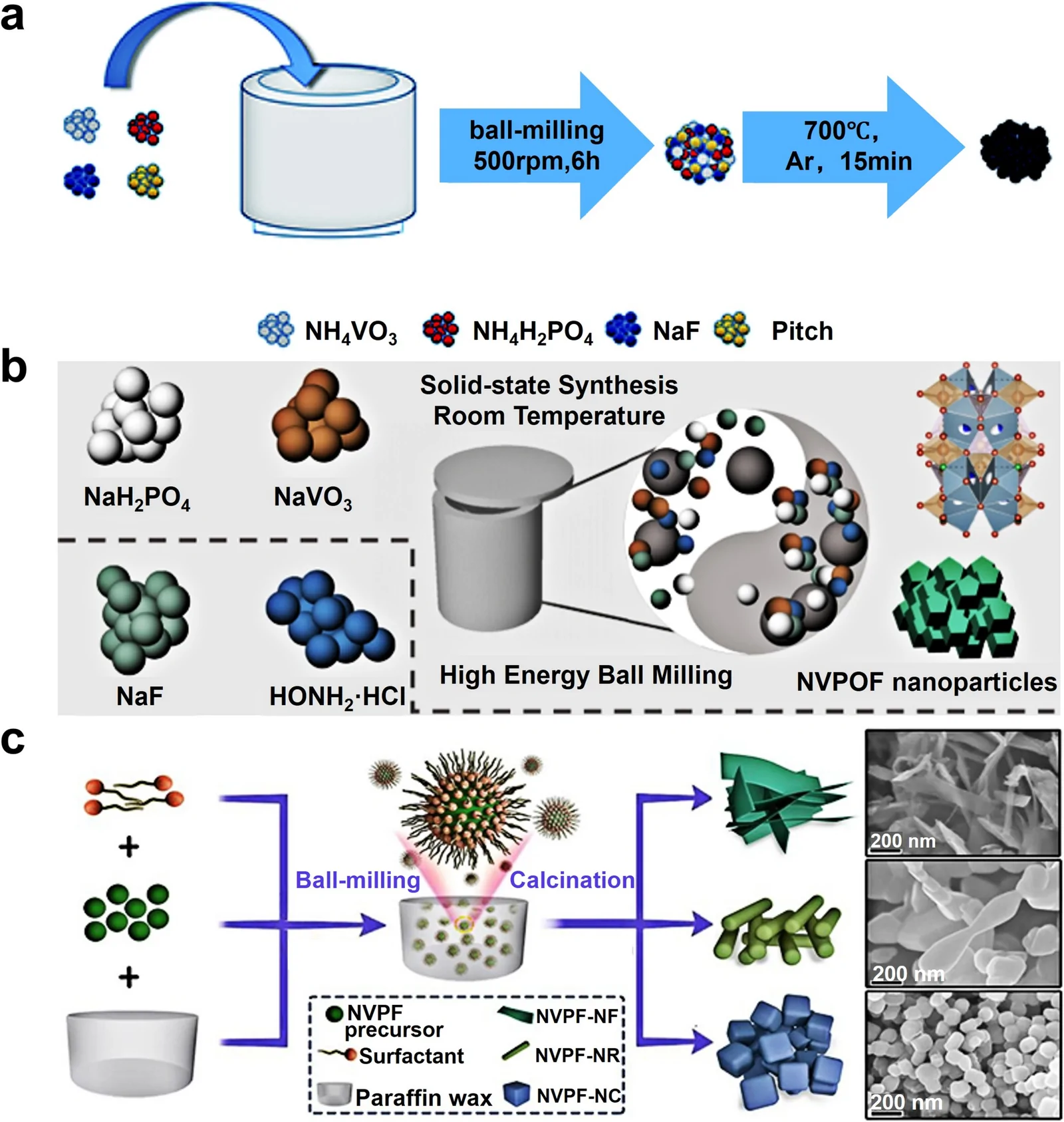
**a** Preparation schematic of NVPF/C composites [72]. Copyright 2019, Royal Society of Chemistry. **b** The mechanochemical synthesis of Na_3_(VOPO_4_)_2_F nanoparticles starting from NaVO_3_ [73]. Copyright 2021, Nature. **c** Schematic synthesis process and SEM images of three NVPF samples [74]. Copyright 2020, Elsevier

In solid-state synthesis, ball milling serves as an effective mechanical activation technique that induces physical and chemical transformations through applied mechanical forces. Using this approach, NVPF/C composites were successfully prepared via a one-step mechanochemically assisted solid-state reaction with asphalt as the carbon source (Fig. 7a) [72]. The synthesis involved short-time calcination at 700 °C under an argon atmosphere. By tuning the asphalt content, composites with different carbon loadings were obtained, among which NVPF/C-2 with 12.14 wt% carbon exhibited optimal electrochemical performance, delivering reversible capacities of 103 and 95 mAh g^−1^ at 0.2C and 10C, respectively, and retaining 91.9% of its capacity after 500 cycles at 5C. Similarly, Shen et al. developed a rapid, solvent-free mechanochemical route for NVOPF synthesis [73]. High-energy ball milling at 600 rpm for 30 min enabled the formation of NVOPF at room temperature (Fig. 7b), demonstrating broad adaptability to vanadium precursors with different valence states. By optimizing the molar ratio of vanadium, phosphorus, and fluorine sources to 1:1.5:1, a high product yield of 94% was achieved. Importantly, this method allows one-step in-situ incorporation of 8 wt% Ketjen Black, producing NVOPF/8%KB nanocomposites with markedly enhanced electronic conductivity. The process has been successfully scaled up to 2 kg per batch, highlighting its strong potential for industrial application.

Beyond compositional control, the introduction of suitable dispersants and surfactants during solid-state synthesis offers an effective route to regulate particle morphology and microstructure. Paraffin can act as a dispersant and, when combined with surfactants such as Span 80, stearic acid, or octadecyl amine (ODA), forms a quasi-water-in-oil microemulsion system that enables the synthesis of individually carbon-coated NVPF nanoparticles (Fig. 7c). By adjusting the type and amount of surfactant, particle architecture can be precisely tailored. The synergistic effects of nanocubic morphology and uniform carbon coating promote Na^+^ ions migration and charge-transfer kinetics, thereby significantly enhancing the cycling stability and rate capability of NVPF-NC [74].

Compared with hydrothermal and sol–gel methods, solid-state synthesis offers notable advantages in terms of low cost, simple processing, and scalability, making it particularly suitable for industrial production. While hydrothermal routes can yield nanoscale particles, they suffer from low productivity and potential safety concerns due to high-pressure conditions. The sol–gel method enables molecular-level mixing and excellent compositional uniformity; however, its high cost and extensive use of organic solvents raise economic and environmental concerns. In contrast, the primary limitations of solid-state synthesis lie in inhomogeneous reactant mixing and relatively large particle sizes, which can restrict electrochemical performance. Consequently, optimizing solid-state synthesis strategies to achieve uniform microstructures and enhanced electrochemical properties remains a key focus for future research.

In summary, these synthesis methods exhibit significant differences in scalability, reproducibility, and industrial relevance. The solid-state method holds the greatest potential for scalable manufacturing owing to a simple process, solvent-free nature, room-temperature mechanochemical synthesis capability, and demonstrated kilogram-scale production. However, limited morphological control and fluorine volatilization at high temperatures remain the primary challenges. The hydrothermal/solvothermal method enables precise morphological regulation to obtain desirable structures such as hollow microspheres and nanoflowers, but industrial application is severely constrained by high-pressure operation, low batch yield, and high solvent consumption. The sol–gel method achieves homogeneous mixing at the molecular level, but the process is complex, requires strict pH control, and involves large amounts of organic solvents. Furthermore, it is generally unsuitable for NVOPF systems, leading to high costs for large-scale production. Therefore, the practical deployment of NVPF and NVOPF relies on the development of continuous solid-state synthesis, improved fluorine stability control, scalable pilot production, and systematic evaluation of full-cell reliability under realistic conditions, thereby bridging the gap between laboratory performance and industrial application. The key characteristics of these methods are summarized in Table 2.

**Table 2:** Comparison of synthesis methods for NVPF and NVOP

Applicability comparison	Hydrothermal/solvothermal	Sol–gel	Solid-state
Key characteristics	Closed system, high pressure, precise morphology control	Molecular-level mixing, pH-sensitive	Simple, scalable, limited morphology control
NVPF	130–200 °C, tunable morphologies	pH ~ 4.2 to suppress F loss and impurities	Ball milling + carbon coating; risk of F loss
NVOPF	120–180 °C, prevents F loss, ideal for hollow structures	Rarely used, F volatilization causes impure phases	Mechanochemical synthesis (room temperature), scalable
Common limitation	Safety concerns, low yield, difficult scale-up	F volatilization, complex control, high cost	Inhomogeneous mixing, large particle size, and F loss at high temperatures
Morphology control	High	Moderate	Low

## Modification Methods

The crystal structures, sodium storage mechanisms, and synthesis strategies of NVPF and NVOPF have been systematically reviewed. Although these materials show considerable promise as cathodes for sodium-ion batteries, their intrinsically low electronic conductivity remains a major obstacle to practical application. Enhancing charge transport properties is therefore essential for improving electrochemical performance [75, 76]. Accordingly, this section focuses on three representative modification strategies, namely surface coating, elemental doping, and nanostructure design, to address these limitations and further optimize the performance of NVPF and NVOPF cathodes. While Sect. 2 elucidates the intrinsic Na^+^ ions diffusion mechanisms within the pristine crystal frameworks, the modification strategies discussed here aim to overcome kinetic limitations through external interventions. These approaches, including surface coating, elemental doping, and nanostructure design, primarily aim to enhance electronic conductivity, modulate the local crystal environment to reduce migration barriers, and shorten solid-state diffusion pathways, respectively.

### Surface Coating

Surface coating is a widely employed strategy to mitigate electrochemical performance degradation of cathode materials arising from interfacial side reactions with electrolytes during charge and discharge. By forming a protective layer on the particle surface, the coating effectively isolates the active material from direct electrolyte contact and enhances structural stability during sodium-ion insertion and extraction. Among various coating materials, carbon-based coatings have attracted considerable attention due to their high electrical conductivity, chemical stability, and low cost. Carbon materials such as amorphous carbon, graphitic carbon, and carbon fibers can uniformly cover the cathode surface to construct continuous conductive networks, suppress interfacial side reactions, and thereby improve cycling stability and rate performance.

**Fig. 8:**
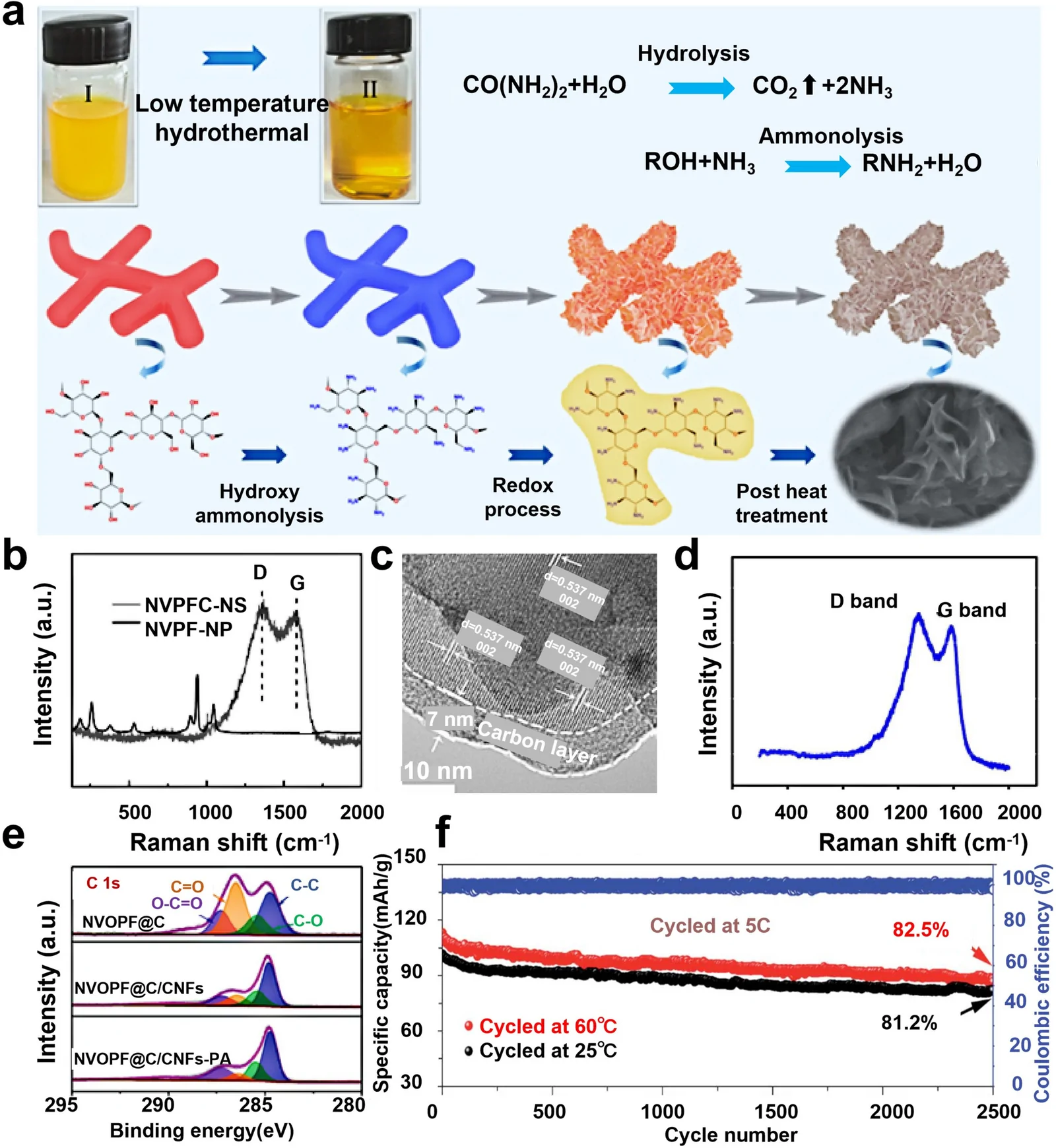
**a** Schematic illustration of the preparation of NVPFC-NS [77]. Copyright 2022, American Chemical Society. **b** Raman spectra of NVPFC-NS and NVPF-NP [77]. Copyright 2022, American Chemical Society. **c** HR-TEM image of NVPF@C nanocomposite and **d** Raman spectrum of carbon [78]. Copyright 2025, Royal Society of Chemistry. **e** C 1*s* XPS spectra of these three different materials [26]. Copyright 2022, Royal Society of Chemistry. **f** Cycling performance for 2500 cycles at 5 C [26]. Copyright 2022, Royal Society of Chemistry

Amorphous carbon coating is one of the most widely used surface modification strategies, as it forms a thin conductive layer that enhances electronic conductivity and mitigates interfacial side reactions [79]. This approach is typically realized by pyrolyzing organic carbon sources such as glucose, sucrose, or starch to achieve uniform carbon deposition [80]. An in-situ amorphous carbon coating on NVPF nanosheets was reported using starch as the carbon source (Fig. 8a) [77]. Raman analysis revealed a high I_D_/I_G_ ratio of 1.06 (Fig. 8b), confirming the disordered and amorphous nature of the carbon layer. Owing to its high defect density and large surface area, the amorphous carbon coating enhances electronic conductivity and facilitates rapid Na ion transport, thereby improving high-rate performance.

A similar effect has been observed in NVOPF systems. Liu et al. [78] constructed an amorphous carbon layer approximately 7 nm on NVOPF nanoparticles (Fig. 8c). This coating not only improves surface electronic conductivity but also serves as a mesoporous carbon matrix that interconnects nanoparticles, forming a three-dimensional conductive network that promotes both electron and ion transport. As a result, the electrode exhibits enhanced rate capability and long-term cycling stability, maintaining high capacity even at high current densities over 3000 cycles. Further performance enhancement can be achieved by combining amorphous carbon with crystalline carbon components. Li et al. deposited an approximately 10 nm amorphous carbon layer on NVOPF particles and confirmed low graphitization degree by XPS, as evidenced by abundant C–O and O–C=O bonds (Fig. 8e) [26]. This amorphous layer synergistically couples with external graphitized carbon nanofibers to form an efficient conductive network, increasing the overall electronic conductivity to about 10^−2^ S cm^−1^. The resulting heterostructure accelerates Na ion diffusion, reduces charge-transfer resistance during de-sodiation ~ 3.6 V, and significantly improves cycling stability at elevated temperatures of 60 °C (Fig. 8f).

**Fig. 9:**
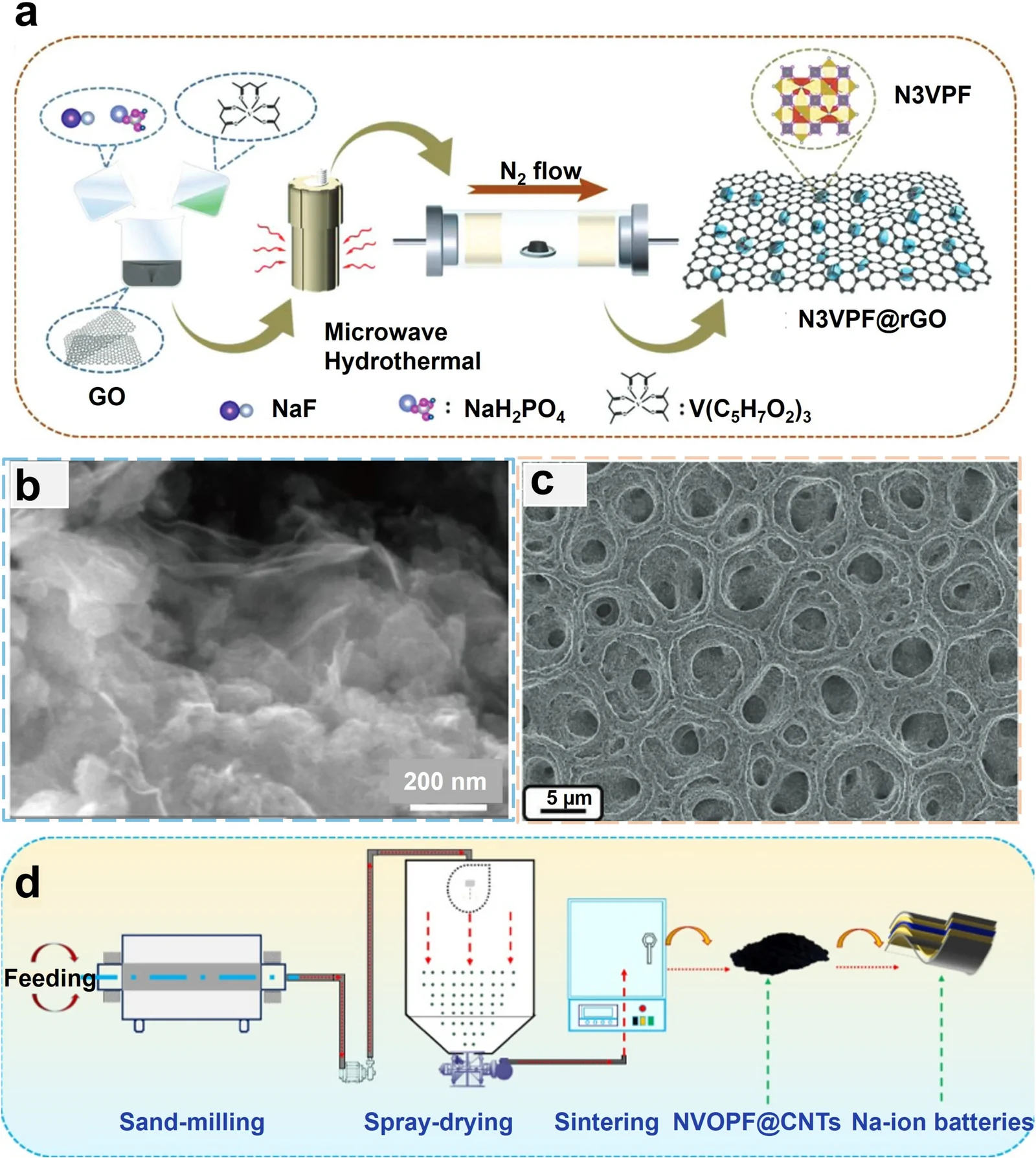
**a** Synthesis procedure of NVPF@rGO [58]. Copyright 2023, Wiley. **b** SEM images of the NVPF@rGO [81]. Copyright 2018, Royal Society of Chemistry. **c** SEM images of bicontinuous ordered 3D porous NVOPF/rGO nanocomposite [82]. Copyright 2019, Wiley. **d** Schematic illustration of the synthesis process of the NVOPF@CNTs composite [83].Copyright 2025, Elsevier

Compared with amorphous carbon, crystalline carbon materials such as reduced graphene oxide and carbon nanotubes offer higher intrinsic conductivity and structural stability. Guan et al. constructed a three-dimensional conductive network by coating reduced graphene oxide onto NVPF nanocubes (Fig. 9a). The highly graphitized carbon framework enhances electronic conductivity, while defect-rich *sp*^2^ and *sp*^3^ carbon domains facilitate charge transport and stabilize the electrode structure during cycling [58]. Similarly, rGO suppresses NVPF particle agglomeration and also constructs a continuous electronic transport network, enhancing the electronic conductivity (Fig. 9b) [81].

In NVOPF systems, reduced graphene oxide also effectively inhibits particle aggregation by forming a 3D porous network that uniformly encapsulates NVOPF nanoparticles with sizes of about 15 nm (Fig. 9c) [82]. This structure provides continuous electron conduction channels while maintaining a low carbon content of only 3.5 wt%, thereby preserving high electrode energy density. Carbon nanotubes represent another effective conductive additive due to the one-dimensional tubular structure, high electrical conductivity, and large aspect ratio. Pi et al*.* introduced carbon nanotubes to construct a three-dimensional conductive framework (Fig. 9d), which accelerates electron transport and improves interparticle connectivity [83]. In addition, carbon nanotubes partially act as reducing agents during heat treatment, promoting the conversion of V^5+^ to V^4+^ and facilitating the formation of phase-pure NVOPF.

Overall, carbon coating strategies play complementary roles in performance enhancement. Amorphous carbon primarily improves ion transport and interfacial conductivity, while crystalline carbon materials (e.g., rGO, CNT) provide long-range electron pathways and suppress particle agglomeration. The rational combination offers an effective route to synergistically enhance the electrochemical performance of NVPF and NVOPF cathode materials. Building upon the foregoing discussion, composite formation with conductive carbon represents a promising strategy for enhancing the overall electrical conductivity of electrode materials. Notably, although carbon coatings enhance the surface electronic conductivity, it does not change the intrinsic electrical conductivity of the material. Furthermore, optimizing the thickness of the carbon layer presents an important direction for future research, as it critically influences both ionic and electronic transport.

### Elemental Doping

Both NVPF and NVOPF exhibit inherently low electrical conductivity, which restricts electrochemical performance. Element doping is an effective strategy for tailoring material properties by introducing trace amounts of metallic or nonmetallic elements into the lattice. Unlike surface coating, which primarily addresses interparticle conductivity and interfacial stability, elemental doping modifies the intrinsic properties of the material. Doping-induced lattice distortion, electronic structure modulation, and valence state adjustment can markedly affect electrical conductivity, mechanical robustness, catalytic behavior, and other key characteristics. The resulting property regulation originates from local structural perturbations and defect formation within the crystal lattice, leading to overall performance enhancement. Appropriate doping enhances ionic and electronic transport by reducing the bandgap and expanding lattice channels, while also improving structural robustness through the suppression of phase transitions and volume changes. However, excessive doping may block diffusion pathways or introduce impurity phases, and the optimal doping level is often narrow and system dependent. The specific impact strongly depends on the dopant species and concentration. This section briefly summarizes the effects of different doping strategies on material properties, with emphasis on applications in NVPF and NVOPF [84, 85]. According to the substitution site of the dopant ion, doping strategies can be classified into vanadium-site doping, sodium site doping, and anion-site doping. Doping at different lattice sites induces distinct structural modifications and property responses.

#### Na-Site Doping

**Fig. 10:**
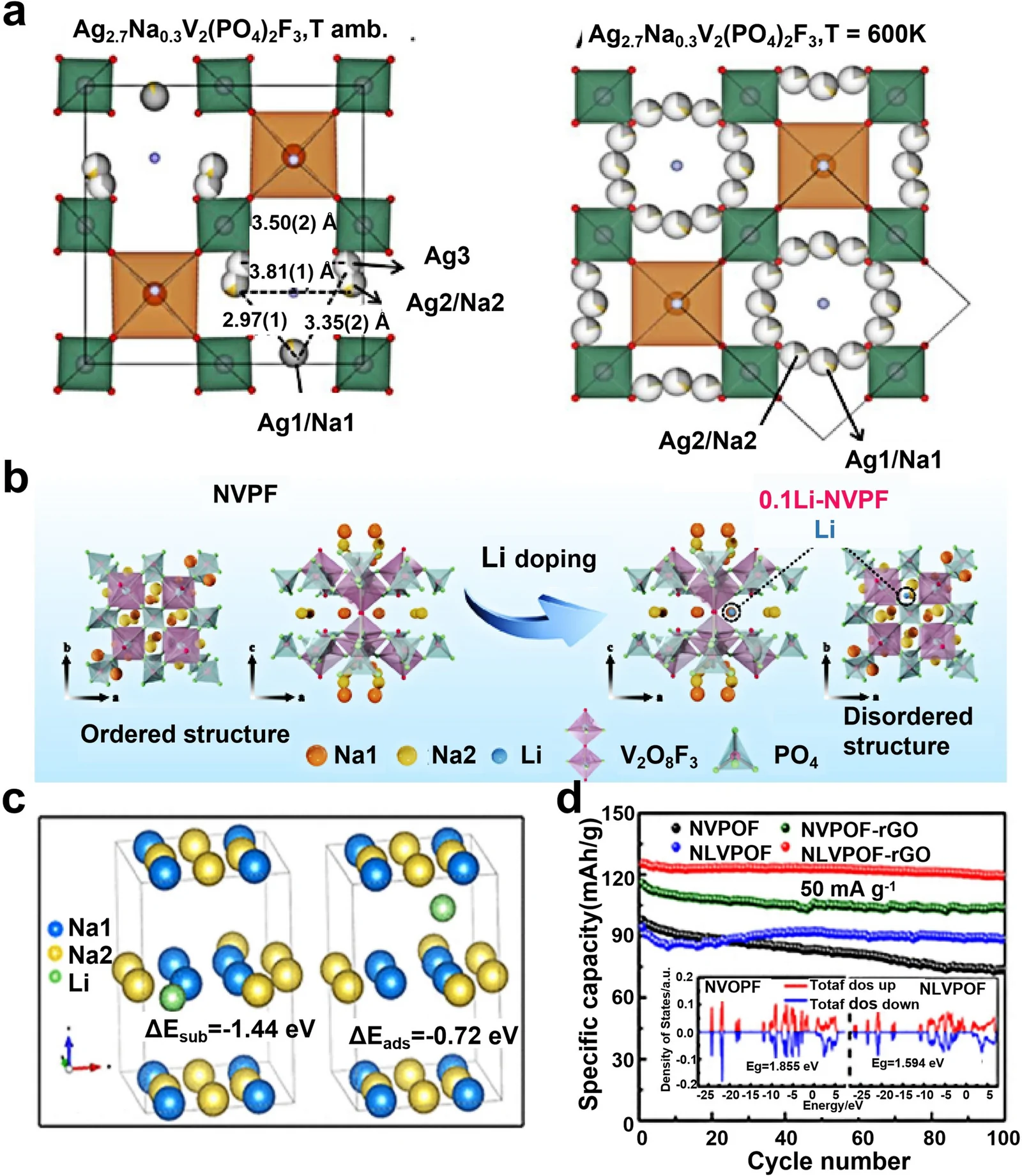
**a** Sodium distribution in Ag_2.71(6)_Na_0.3(2)_V_2_(PO_4_)_2_F_3_ [86]. Copyright 2016, Royal Society of Chemistry. **b** Schematic illustration of NVPF and 0.1Li-NVPF [30]. Copyright 2025, Wiley. **c** Charge density difference diagrams [87]. Copyright 2020, American Chemical Society. **d** Galvanostatic charge/discharge performance of samples [87]. Copyright 2020, American Chemical Society

Na-site doping modulates the properties of NVPF and NVOPF materials by introducing other ions to replace partial sodium sites. The mechanism lies in enhancing electronic conductivity by forming mixed valence states of vanadium and optimizing the Na^+^ ions migration pathways by altering the local electrostatic environment and Na^+^ ions/vacancy ordering, thereby significantly improving the rate performance of the material. Simultaneously, the dopant ions act as structural pillars, suppressing volume changes and phase transitions during charge–discharge cycles, thereby enhancing structural stability and cycle life. M. Bianchini et al*.* introduced silver into the NVPF framework via Ag^+^/Na^+^ ions exchange [86]. The study successfully synthesized nearly fully-doped Ag_3_V_2_(PO_4_)_2_F_3_ and partially-doped Ag_2.7_Na_0.3_V_2_(PO_4_)_2_F_3_. Silver doping preserved the crystalline framework, and also increased orthorhombic distortion, and elevated the cation disorder transition temperature within the structure (Fig. 10a). Furthermore, silver incorporation enables the material to maintain high structural stability at high temperatures. Similarly, Na_2.9_Li_0.1_V_2_(PO_4_)_2_F_3_ (0.1Li-NVPF) was constructed by introducing Li^+^ for Na-site doping (Fig. 10b) [30]. Li^+^ preferentially occupies the Na1 sites, leading to lattice contraction and charge redistribution, thereby reducing the electron density around F^−^ ions. Simultaneously, the introduction of Li^+^ weakens the Coulomb repulsion between adjacent Na^+^ ions through electrostatic shielding. This significantly reduced the Na^+^ migration energy barrier by disrupting the ordered Na^+^ ions/vacancy arrangement, thereby promoting faster diffusion kinetics. Furthermore, Li doping disrupts the ordered arrangement of Na^+^ ions, promoting Na^+^ ions migration. Li doping is also an effective strategy for optimizing NVOPF performance. Unlike NVPF, theoretical calculations indicate that Li^+^ preferentially occupies the Na2 site, forming a more stable structure, reducing the bandgap, and enhancing electronic conductivity (Fig. 10c) [87]. Introducing Na vacancies and interstitial Li through Li doping can enhance Na^+^ ions migration and increase the number of Na storage sites. The battery assembled with Li doping combined with reduced graphene oxide modification retained 89.4% of the capacity after 500 cycles (Fig. 10d).

In summary, although Na-site doping serves as a universal strategy for optimizing both NVPF and NVOPF materials, the specific mechanism depends on the structure of the material and the dopant ions. For instance, Li^+^ ions tend to occupy the Na1 site in NVPF, promoting ion migration through electrostatic shielding effects and disruption of Na^+^ ions ordering. In contrast, within NVOPF, Li^+^ ions preferentially occupy the Na2 site, which reduces the bandgap to enhance electronic conductivity and increases ion migration pathways by introducing Na vacancies. These results indicate that different doping designs based on the structural characteristics of different material systems are key to improving electrochemical performance.

#### Vanadium-Site Doping

**Fig. 11:**
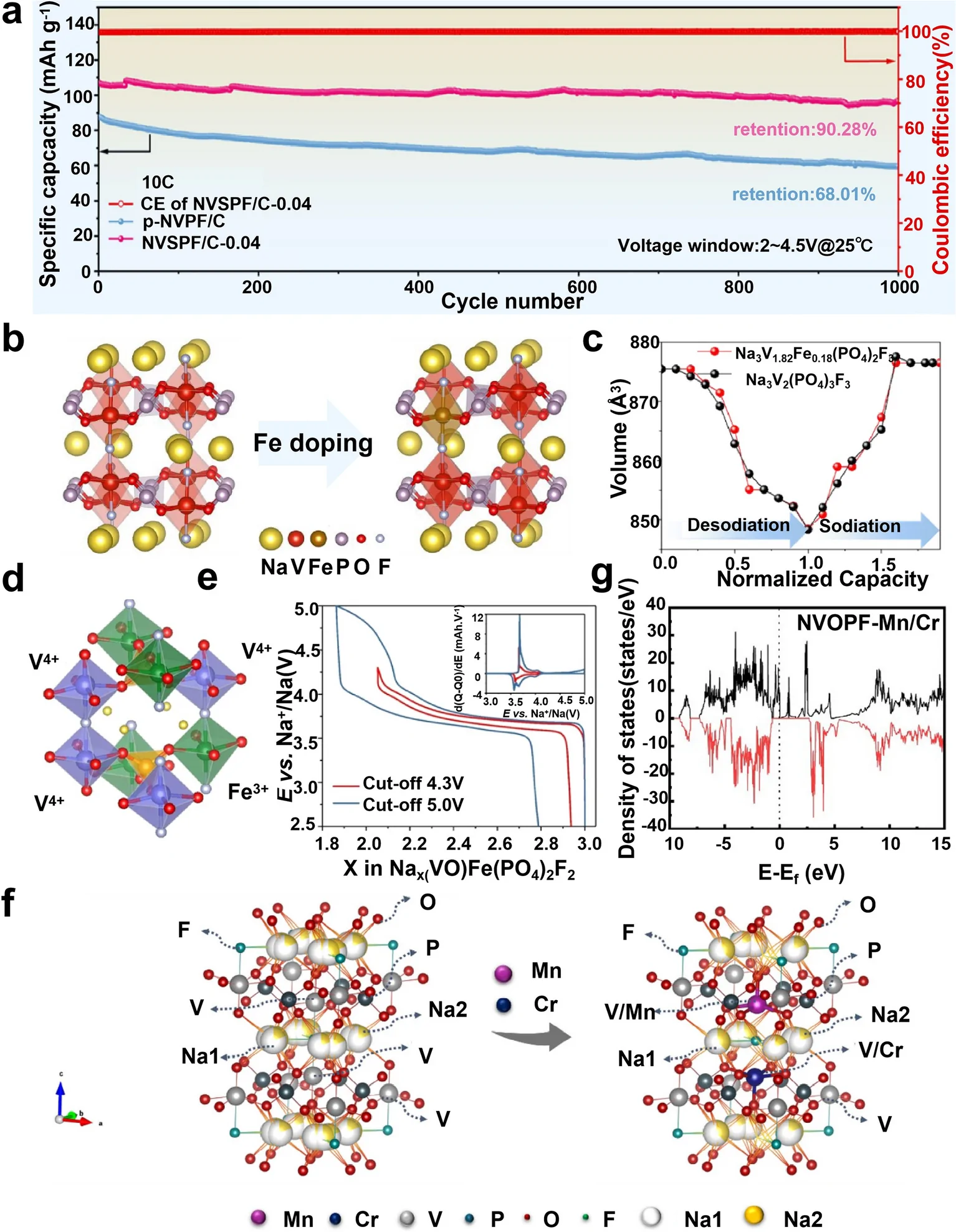
**a** Electrochemical performance of p-NVPF/C and NVSPF/C-0.04 [91]. Copyright 2025, American Chemical Society. **b** Crystal structures of Na_3_V_2_(PO_4_)_2_F_3_ and Na_3_V_1.75_Fe_0.25_(PO_4_)_2_F_3_ [92]. Copyright 2018, Royal Society of Chemistry. **c** Unit cell volume variations of undoped NVPF and Fe-doped NVPF [92]. Copyright 2018, Royal Society of Chemistry. **d** Crystal structure of Fe-doped NVOPF [93]. Copyright 2019, American Chemical Society. **e** Charge/discharge curve of Na_3_(VO)Fe(PO_4_)_2_F_2_ [93]. Copyright 2019, American Chemical Society. **f** Crystal structure of NVOPF and NVMC-95 [66]. **g** DOS of NVOPF-Mn/Cr co-doping [66]. Copyright 2025, Wiley

Vanadium-site doping enhances the structural stability of the material while simultaneously improving both electronic conductivity and ionic transport [88–90]. The electrochemical performance of NVPF was optimized by partially substituting V^3+^ with Sc^3+^ [91]. Sc doping stabilizes the crystal structure and suppresses the volume change during the charge–discharge cycle. Meanwhile, it reduces the lattice parameters, thereby promoting the diffusion rate of sodium ions. Additionally, Sc doping increased the graphitization degree of the carbon coating, enhanced electronic conductivity, and reduced charge-transfer impedance. When the Sc doping amount is 0.04, the sample exhibits excellent electrochemical performance, indicating the effective role of Sc doping in regulating the ionic conductivity and structural stability of the material (Fig. 11a). However, the high cost of Sc would increase commercial application expenses. Therefore, selecting a cost-effective doping element is of critical importance. Park et al*.* achieved dual objectives of performance enhancement and cost reduction by partially substituting vanadium (V) with inexpensive iron (Fe) in the NASICON-type cathode material NVPF [92]. Fe doping promotes efficient utilization of the V^3+^/V^4+^ redox pair and enhances Na^+^ ions diffusion coefficient by introducing an intermediate-phase buffer layer and reducing the material bandgap (Fig. 11b, c). Fe doping has also been applied to NVOPF. The material Na_3_(VO)Fe(PO_4_)_2_F_2_ was successfully synthesized (Fig. 11d) by partially replacing V^4+^ ions with Fe^3+^ ions [93]. The random distribution of Fe^3+^ ions and V^4+^ ions effectively suppresses the ordering of Na^+^ vacancies. This reduced the formation energy barrier for Na^+^ ions defects, thereby enhancing the migration rate of Na^+^ ions within structural channels and improving the ionic conductivity. Although the Fe^3+^/Fe^4+^ redox pair has no electrochemical activity in the high-potential region (> 4.3 V) (Fig. 11e), Fe^3+^ ions doping reduces electrode polarization and improves rate performance. Furthermore, the Fe^3+^/Fe^2+^ reaction can be activated in the low-potential region (~ 1.5 V), enabling the insertion of approximately 0.5 Na^+^ ions and enhancing reversible capacity. Bimetallic co-doping demonstrates strong potential for optimizing material electronic structure and ionic transport kinetics. Through a synergistic co-doping strategy of Mn and Cr (Fig. 11f), the NVOPF cathode material achieved performance enhancement [66]. The introduction of Mn^3+^ ions primarily enhances the electronic conductivity (DFT calculations show the bandgap drastically reduced from 2.15 to 0.12 eV) (Fig. 11g), while Cr^3+^ ions doping effectively strengthens structural integrity and improves Na^+^ ions transport kinetics. The random Co-doping of two ions at the V site led to moderate lattice distortion, thereby expanding the migration path of Na^+^ ions. At the same time, it also inhibited the ordered arrangement of vacancies of Na^+^ ions during the charge and discharge cycles. The results show that the specific capacity of NVMC-95 material at 20C is 87 mAh g^−1^. In summary, NVPF doping mainly focuses on optimizing structural stability and dynamics, while NVOPF has broader potential in enhancing electron/ion cotransport capabilities due to the inherent tunable electronic structure.

The efficacy of vanadium-site doping is further validated in full-cell configurations. Cu^2+^-doped NVPF (NVC_0.05_PF@C) paired with hard carbon (HC) achieves an energy density of 460 Wh kg^−1^ (based on cathode mass) and retains 96% of its capacity after 1000 cycles at 10C, demonstrating that the “anchor effect” induced by larger Cu^2+^ ions effectively stabilizes the NVPF framework under high-rate conditions [94]. In contrast, Mn/Cr co-doped NVOPF (NVMC-95) delivers a slightly lower energy density of ~ 412 Wh kg^−1^ in full cells but exhibits superior capacity retention of 97% after 100 cycles at 0.2C, reflecting the intrinsic structural reversibility of NVOPF and the synergistic role of Mn and Cr in enhancing electronic conductivity (bandgap reduced from 2.15 to 0.12 eV by DFT calculations) while suppressing lattice volume changes [66]. These complementary findings underscore that NVPF benefits more from doping-induced lattice expansion and electronic bandgap narrowing to overcome its intrinsically lower conductivity, whereas NVOPF leverages co-doping to further enhance its already favorable Na^+^ diffusion kinetics and structural robustness, offering distinct pathways for optimizing each material in practical sodium-ion full cells.

#### Anion-Site Doping

**Fig. 12:**
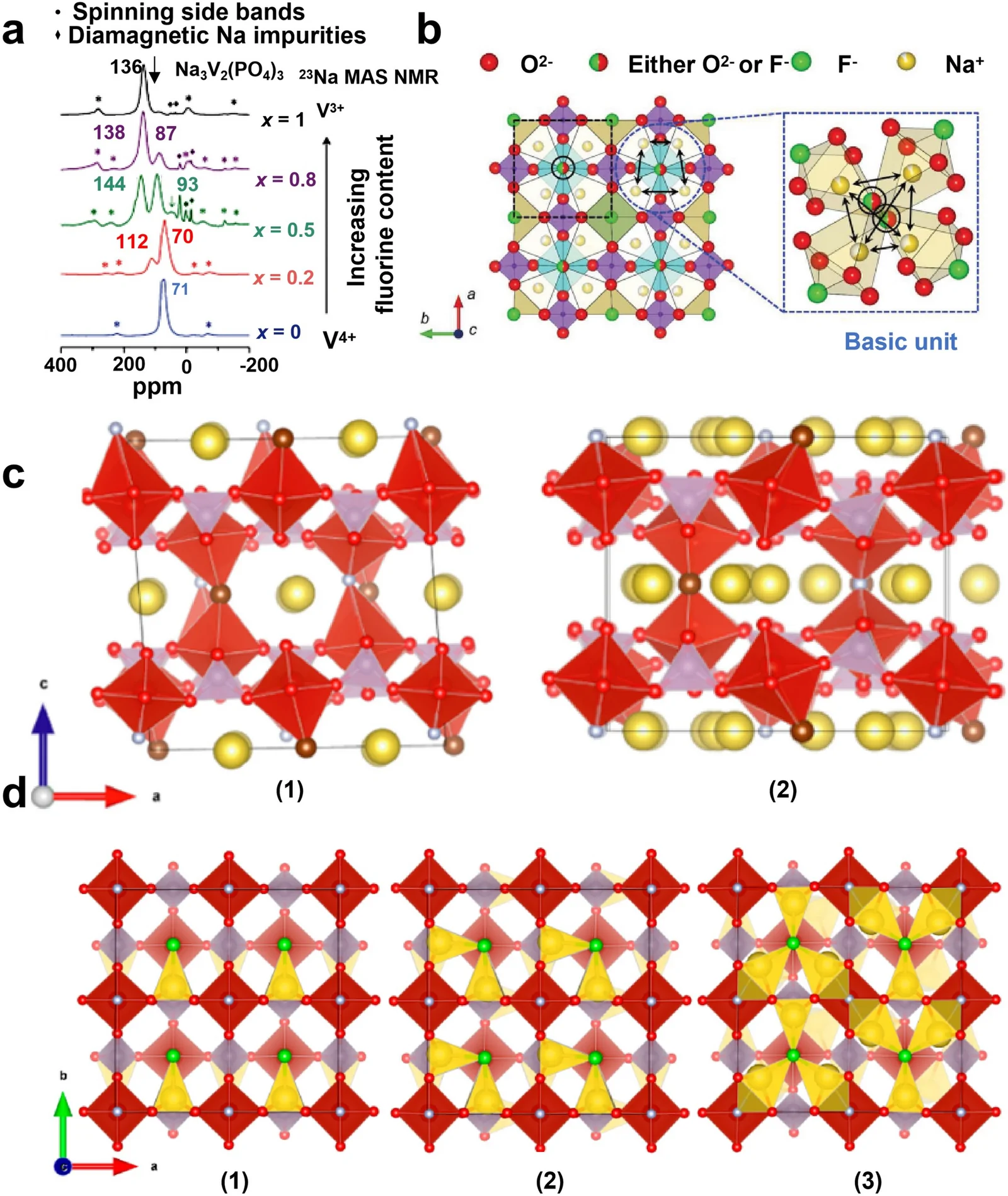
**a** ^23^Na MAS NMR spectra of the Na_3_(VO_1−*x*_PO_4_)_2_F_1+2*x*_ (*x* = 0.0, 0.2, 0.5, 0.8, and 1.0) samples at 15 kHz [39]. Copyright 2014, Wiley. **b** Intra-unit Na^+^ ions repulsion in the Na layer of Na_3_(VO_1−*x*_PO_4_)_2_F_1+2*x*_ (0 ≤ *x* ≤ 1) [39]. Copyright 2014, Wiley. **c** Crystal structures of (1) NaV_2_(PO_4_)_2_F_0.5_Br_0.5_O_2_ and (2) Na_3_V_2_(PO_4_)_2_F_0.5_Br_0.5_O_2_, the brown atoms are Br [96]. Copyright 2014, American Chemical Society. **d** Crystal Structures of (1) NaV_2_(PO_4_)_2_FCl_2_, (2) Na_2_V_2_(PO_4_)_2_FCl_2_, (3) Na_3_V_2_(PO_4_)_2_FCl_2_, the green atoms are Cl [96]. Copyright 2014, American Chemical Society

Anion-site doping primarily modulates material properties by substituting O^2−^ or F^−^ ions within the NVPF/NVOPF framework. In 2014, Park et al*.* systematically controlled the fluorine (F) content (0 ≤ *x* ≤ 1) in Na_3_V_2_O_2(1−*x*)_(PO_4_)_2_F_1+2*x*_ to achieve continuous regulation of vanadium (V) valence states (V^3+^/V^4+^/V^5+^) (Fig. 12a), thereby establishing a complete solid-solution system [39]. The introduction of fluorine alters the anion composition within the crystal structure, and also enhances the average operating voltage. At the atomic level, replacing O^2−^ with F^−^ modify the ligand field around vanadium; the higher electronegativity of F^−^ lowers the V 3d orbital energy levels through a stronger inductive effect, which elevates the redox potential while narrowing the bandgap and enhancing electronic conductivity. Furthermore, the insertion/extraction mechanism and phase transition behavior of sodium can be affected by modulating the electrostatic repulsion between Na^+^ ions in the sodium layer (Fig. 12b). Similarly, the stable regulation of mixed V^3+^/V^4+^ oxidation states was achieved by controlling the oxygen (O)/fluorine (F) ratio in NVOPF materials [95]. This material essentially achieves anion-site doping to form a solid solution. This adjustment of the O/F ratio influences the material electronic structure, such as average oxidation state and charge distribution.

However, another more direct regulatory strategy is to alter the local coordination environment through ion substitution. Goodenough et al*.* focused on replacing the “dangling” O^2−^ ions coordinated to vanadium in NVOPFs with less electronegative halide anions (e.g., Cl^−^ or Br^−^) (Fig. 12c, d). Theoretical calculations indicate that this anion doping effectively weakens the covalent nature of the V-X (X = Cl, Br) bond and reduces the Coulombic attraction between Na^+^ and the framework anion [96]. Consequently, the desorption voltage for the third Na^+^ ion is significantly lowered from the previously excessive 5.3 V to within the electrolyte stability window (~ 4.8 V). Among these, Na_3_V_2_(PO_4_)_2_FCl_2_, formed by complete Cl^−^ substitution of O^2−^, is considered the most promising material. It can reversibly insert/extract three Na^+^ ions, raising the theoretical energy density to 758 Wh kg^−1^, but computational analysis also confirms the structural stability during charge–discharge cycles. Furthermore, the voltage plateaus can be optimized by controlling anion type and ratio (e.g., NVPF_0.5_Br_0.5_O_2_). Anion chemistry plays a pivotal regulatory role in polyanionic cathode materials that extends far beyond mere structural support. The essence depends on systematically determining the intrinsic electrochemical properties of materials through modifying the local electronic structure and crystal field environment. Current research has confirmed that adjusting the oxygen/fluorine ratio or introducing halogen ions such as Cl^−^ and Br^−^ ions can effectively control the valence states and electronic configurations of transition metals, thereby influencing the crystal field environment, Na^+^ ions migration energy barriers, and phase transition pathways. However, complete substitution or the use of bromine may still pose synthesis challenges and energy density losses, suggesting the need for more refined doping strategy design. Specifically, partially substituting PO_4_^3−^ with SiO_4_^4−^ ions, which possesses lower electronegativity and a larger ionic radius, firstly regulates the redox energy levels of transition metals through a stronger inductive effect [97].

In vanadium-based systems, this effectively narrows the V^4+^/V^5+^ energy level gap, enabling the activation of high-voltage plateaus to enhance energy density [98]. In iron/manganese-based systems, it significantly optimizes the reversibility of the Fe^2+^/Fe^3+^ and Mn^2+^/Mn^3+^ redox reactions [90]. Secondly, lattice perturbations induced by this substitution fundamentally reduce the Na^+^ migration barrier, a key mechanism behind enhanced rate performance as jointly verified by DFT calculations and GITT tests. More importantly, the chemical nature of anions directly dictates electrode/electrolyte interface stability. The introduction of SiO_4_^4−^ or SO_4_^2−^ ions alters surface electron cloud distribution, impacting the composition and formation kinetics of the CEI layer, with pronounced reductions in charge-transfer resistance and interface film impedance clearly observed [99]. Thus, the essence of anion chemistry lies in its ability to simultaneously regulate three critical dimensions: bulk electronic structure, ionic migration channels, and surface/interface reaction kinetics, constituting an intrinsic optimization strategy that transcends the limitations of conventional cation doping. Despite notable achievements, the field still possesses substantial untapped potential. Beyond O^2−^, F^−^, Cl^−^, and Br, the impacts of doping with other anions such as S^2−^ and N^3−^ ions remain insufficiently understood. Future research should broaden the scope of anion doping and integrate advanced operando characterization techniques with theoretical calculations to provide deeper insights into the "composition–structure–performance" relationships. Such efforts will offer theoretical guidance and innovative design concepts for developing the next generation of high-performance cathode materials. To comprehensively understand the effects of different types of dopants and substitution points on electrochemical behavior, Table 3 summarizes a series of ion doped NVPF and NVOPF systems, including specific capacity and long-term cycling stability.

**Table 3:** Summary of ion-doped NVPF and NVOPF performance

Materials	Dopant element	Dopant site (s)	Specific capacity (mAh g^−1^)	Cycle performance	References
0.1Li-NVPF/CNTs	Li	Na	114.69 (1 C)	64.1% (30,000 cycles) at 10C	[30]
NVSPF/C-0.04	Sc	V	126 (0.2 C)	90.28% (1000 cycles) at 10C	[91]
HE-NVPF	Ca, Mg, Al, Cr, Mn	V	118.5 (0.1 C)	80.4% (2000 cycles) at 20C	[100]
Na_3_V_1.95_Nb_0.05_(PO_4_)_2_O_2_F/rGO	Nb	V	106 (0.5 C)	60.8% (500 cycles) at 10C	[67]
NVPF-Zr-0.02/NC	Zr	V	119.2 (0.5 C)	90.2% (1000 cycles) at 20C	[101]
NKVPF	K	V	128.8 (0.2 C)	60.2% (5000 cycles) at 10C	[102]
NVPFCa-0.05/C	Ca	V	125 (0.1 C)	70% (1000 cycles) at 10C	[103]
NVPOFSi_0.05_	SiO_4_^4−^	PO_4_^3−^	126 (0.5 C)	95.6% (1000 cycles) at 10C	[98]
NVPF–Mg_0.5_	Mg	V	126.8 (0.1 C)	70% (1000 cycles) at 20C	[104]
NVSnPF-0.07@rGO	Sn	V	126.5 (0.5 C)	92.3% (600 cycles) at 10C	[105]

### Nanoscale Strategy

As mentioned earlier, both surface coating and elemental doping can enhance material conductivity. Typically, reducing material particle size to the nanoscale is another approach to improving conductivity. Consequently, current research focuses on developing NVPF and NVOPF with nanostructures.

**Fig. 13:**
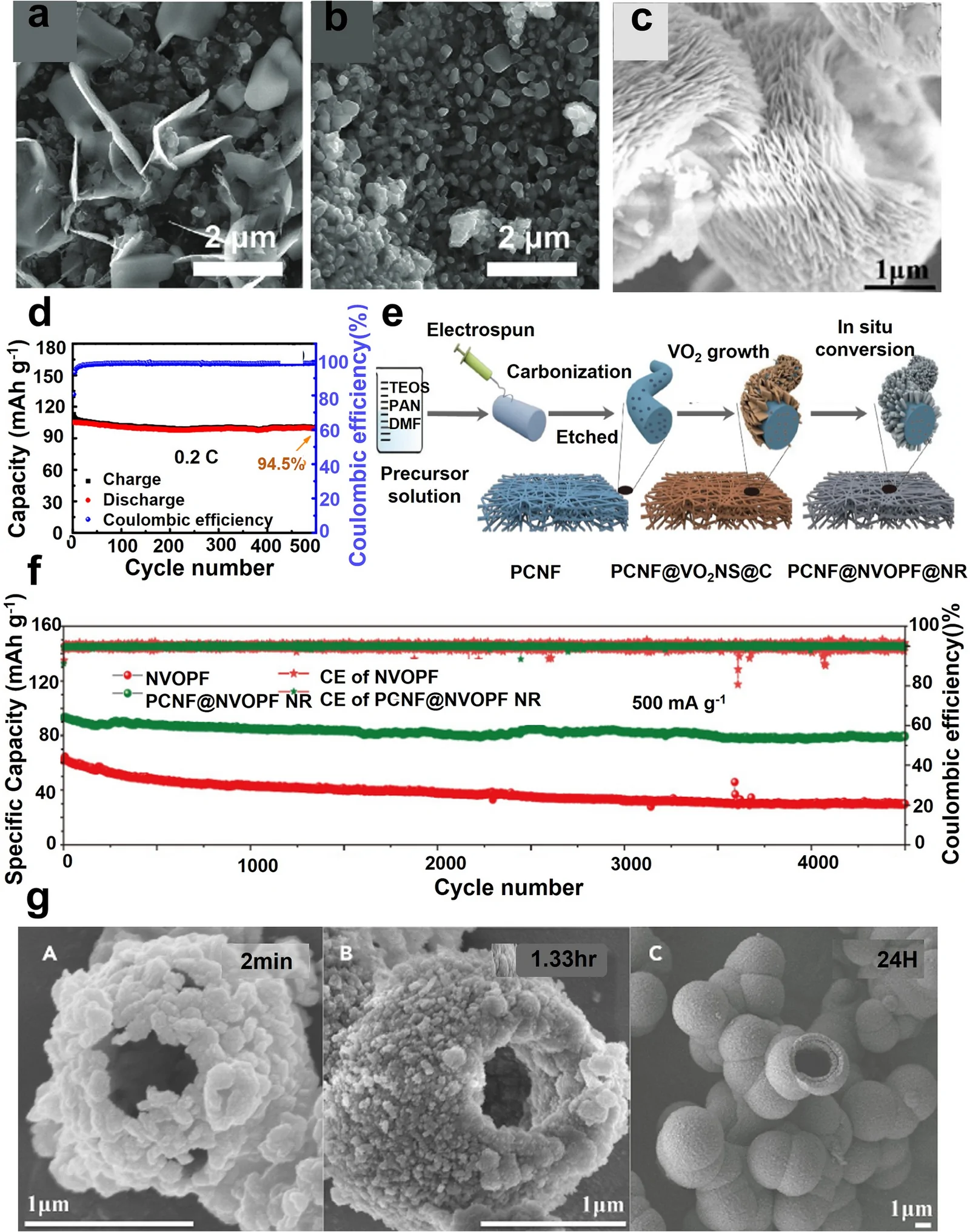
SEM images of **a** m-NVPF and **b** n-NVPF [106]. Copyright 2020, Wiley. **c** SEM images of NVPF [107]. Copyright 2016, Royal Society of Chemistry. **d** the cycling performance and Coulombic efficiency at current rates of 0.2C [107]. Copyright 2016, Royal Society of Chemistry. **e** Schematic illustration of VO_2_ NS arrays and NVOPF NR arrays on PCNF [65]. Copyright 2023, Springer. **f** Cycling stability for 4500 cycles at 500 mA g^−1^ [65]. Copyright 2023, Springer. **g** Morphology evolution of NVPOF microspheres at different reaction time intervals [108]. Copyright 2018, Elsevier

It is well known that reducing particle size to the nanoscale significantly enhances the rapid transport of ions and electrons within electrode materials. Furthermore, this nanostructuring strategy increases the specific surface area of the electrode, exposing more active sites for electrochemical reactions, which can accelerate reaction kinetics and improve specific capacity. Based on these advantages, Song et al*.* incorporated graphene to suppress NVPF grain growth, downsizing the material from the micrometer scale (≈2 µm) (Fig. 13a) to the nanoscale (≈300 nm) (Fig. 13b), thus obtaining nanostructured n-NVPF [106]. This nanostructuring engineered multiple synergistic benefits: first, it reduces Na^+^ ions diffusion paths; second, it increases the specific surface area of the electrode material, providing more electrochemical reaction sites; third, graphene constructs a highly efficient electronic conduction network, compensating for the limitations of material conductivity. NVPF nanoflower architectures were synthesized via a pH-controlled hydrothermal method under weakly acidic conditions (pH = 2.60). The nanoflowers are composed of self-assembled nanosheets with a uniform thickness of approximately 10 nm, forming flower-like structures with diameters of about 8 μm and thicknesses of 4 μm (Fig. 13c). This ordered hierarchical architecture provides a high specific surface area and abundant Na^+^ ions transport pathways, while enabling effective electrolyte penetration through the inter-sheet gaps. Compared with conventional nanoparticle morphologies, the nanoflower structure significantly shortens Na^+^ ions diffusion distances and improves reaction kinetics. As a result, the assembled cells exhibit excellent cycling stability, with a capacity retention of 94.5% after 500 cycles (Fig. 13d) [107].

A highly ordered NVOPF nanorod array architecture was constructed on porous carbon nanofibers (PCNF) through an in-situ conversion strategy (Fig. 13e) [65]. In this configuration, nanorods with widths of approximately 30 nm serve as the fundamental building units and are vertically aligned on a flexible conductive substrate, forming a three-dimensional conductive network with a high specific surface area of 30.9 m^2^ g^−1^. This nanostructured design effectively suppresses active material agglomeration and enhances solid–liquid interfacial contact between the electrode and electrolyte. Meanwhile, the PCNF framework provides continuous electronic conduction pathways and mechanical reinforcement. Benefiting from these synergistic effects, the electrode delivers outstanding long-term cycling performance, maintaining 87.6% of its initial capacity after 4500 cycles (Fig. 13f). In addition, a multi-shell NVOPF microsphere architecture was developed using primary nanoparticles of approximately 20 nm as building blocks, which self-assemble into hollow microspheres with hierarchical porosity (Fig. 13g) [108]. Distinct from the temperature-driven morphological evolution discussed in the context of synthesis methods, this architecture is specifically engineered as a nanostructuring strategy to optimize electrochemical performance. This multilevel nanostructure increases the specific surface area and exposes abundant electrochemically active sites. More importantly, it shortens Na^+^ ions diffusion pathways and effectively buffers volume variations during repeated Na^+^ ions insertion and extraction, thereby enhancing the structural stability of the electrode.

Nanostructure design yields distinct benefits for NVPF and NVOPF in full cells. The hierarchical hollow architecture of NVOPF@KB enables an NVOPF@KB||HC full cell to deliver 122.3 mAh g^−1^ at 0.1 C and retain 88.8% capacity after 100 cycles at 1C, leveraging the hollow structure to buffer volumetric changes [109]. For NVPF, the synergistic combination of rGO wrapping and truncated morphology in NVPF@rGO||HC full cells results in stable operation at high rates, with 80% capacity retention after 300 cycles at 5C [110]. Notably, the two‑dimensional Na^+^ diffusion pathways engineered in NVPOF‑80 (a derivative of NVOPF) through mild structural perturbation enable an NVPOF‑80||3DSe full cell to achieve an ultrahigh energy density of 458.3 Wh kg^−1^ at 1C and 313.8 Wh kg^−1^ at 10C [111], showcasing the superior rate capability that can be unlocked when the intrinsic diffusion kinetics of NVOPF are fully exploited.

Overall, nanostructure engineering plays a crucial role in enhancing the electrochemical performance of sodium-ion battery cathode materials. Rational nano-architectural design enables NVPF and NVOPF to effectively overcome intrinsic limitations, including low electronic conductivity and sluggish Na^+^ ions diffusion kinetics, leading to improved rate capability and cycling stability.

## Interface Stability and Electrolyte Optimization

### High-Voltage-Induced Interfacial Reactions and Thermal Failure

The interfacial stability of NVPF and NVOPF as cathode materials for sodium-ion batteries is a critical factor influencing the cycle life, capacity retention rate, and safety performance. In NVPF, one V atom coordinates with six F^−^ ions, forming [VF_6_] octahedra. This lowers the energy levels of the vanadium 3d orbitals, reducing the Fermi level [112, 113]. Consequently, NVPF exhibits a high redox potential of approximately 4.1 V [114]. The voltage surpasses the thermodynamic stability window (~ 3.5–3.7 V vs. Na^+^/Na) of conventional carbonate-based electrolytes [115–117]. Because the interface reaction is thermodynamically driven, the difference in voltage windows is prone to thermal runaway. During thermal runaway, the reaction proceeds moderately due to the stable crystal structure and low tendency to release oxygen. It is primarily characterized by electrolyte decomposition rather than cathode degradation. The electrolyte decomposition produces gases, primarily CO_2_ (42%), along with a considerable amount of incompletely decomposed electrolyte solvent (e.g., DMC, 15%), and small amounts of H_2_ (15%) and CO (10%) [118]. Meanwhile, trace amounts of HF were detected, indicating that the decomposition reaction is mainly dominated by the thermal decomposition of the electrolyte. Different triggering mechanisms can have a significant influence on failure behavior. Overcharging results in the highest total gas volume and proportion of hydrogen due to the additional energy input. In contrast, low-energy triggers such as external short circuits result in relatively mild reactions [118].

Overall, NVPF batteries demonstrate comparatively low thermal runaway temperatures and a narrow flammable gas explosion range. In contrast, in NVOPF, two O^2−^ ions substitute for two F^−^ ions in the [VF_6_] octahedra, forming a [VO_5_F] mixed-coordinated octahedron [119]. The electronegativity of O^2−^ ions is significantly weaker than that of F^−^, and the electron cloud distribution is also different [120]. This causes the Fermi level of the material to shift upward, which is macroscopically reflected as an average operating voltage of approximately 3.8 V. The degradation mechanisms of NVOPF cathode materials in sodium-ion batteries remain inadequately elucidated. One possible reason for the failure of the positive electrode is a voltage drop of ~ 0.2 V. This reduction shifts the cathode potential from “substantially exceeding” to “marginally surpassing” the electrochemical stability window of the electrolyte. Although oxidative decomposition can still occur, the thermodynamic driving force is reduced, and the kinetic processes become more controlled. Simultaneously, the electrolyte causes the dissolution of the passivation layer (Al_2_O_3_) on the surface of the aluminum current collector, triggering severe pitting corrosion at high voltage [115, 121]. This disrupts the conductive network and the corrosion products further contaminate the electrode interface, leading to electrode failure. In NVOPF, F^−^ ions are more likely to dissolve into the electrolyte and combine with H^+^ in the electrolyte to form HF, which is highly corrosive. This in-situ-generated HF continuously attacks the surface crystal structure of the cathode material [122–124]. This accelerates the dissolution of active vanadium species, resulting in direct capacity loss, and degrades the framework, causing structural collapse, phase transitions, and irreversible capacity fading.

Further exploration in this area can be conducted to help researchers gain a better understanding of the failure mechanism. Specifically, for NVPF, the high operating voltage triggers continuous oxidative decomposition of carbonate-based electrolytes, generating a thick and resistive CEI layer that progressively increases interfacial impedance and accelerates capacity decay. For NVOPF, while the lower voltage reduces the thermodynamic driving force for electrolyte oxidation, the partial substitution of F^−^ by O^2−^ ions renders the structure more susceptible to fluorine dissolution, forming HF that corrodes the cathode surface and causes vanadium loss. These distinct chemical processes, namely CEI overgrowth in NVPF and HF-induced corrosion in NVOPF, directly dictate the respective cycling stability and performance decay pathways, highlighting the need for tailored electrolyte and interface engineering strategies for each material system.

### CEI Driven Interfacial Failure and Capacity Decay

The cycling stability and coulombic efficiency of NVPF and NVOPF at high voltages depend on the stability of the CEI. As mentioned in the section on failure mechanisms, the NVPF cathode provides a substantial thermodynamic driving force due to the high voltage, which forces the electrolyte solvents (such as EC and DEC) and salt anions (PF_6_^−^) to undergo oxidative decomposition. This results in the formation of CEI film during the charging and discharging process of NVPF [116, 125]. This layer has high impedance and cannot effectively isolate electrons, leading to continued decomposition reactions at the interface between the CEI and the electrolyte. Therefore, the CEI film continues to thicken, the internal resistance increases, and the capacity decreases rapidly.

### Electrolyte Design Strategies for High-Voltage Interfacial Stability

**Fig. 14:**
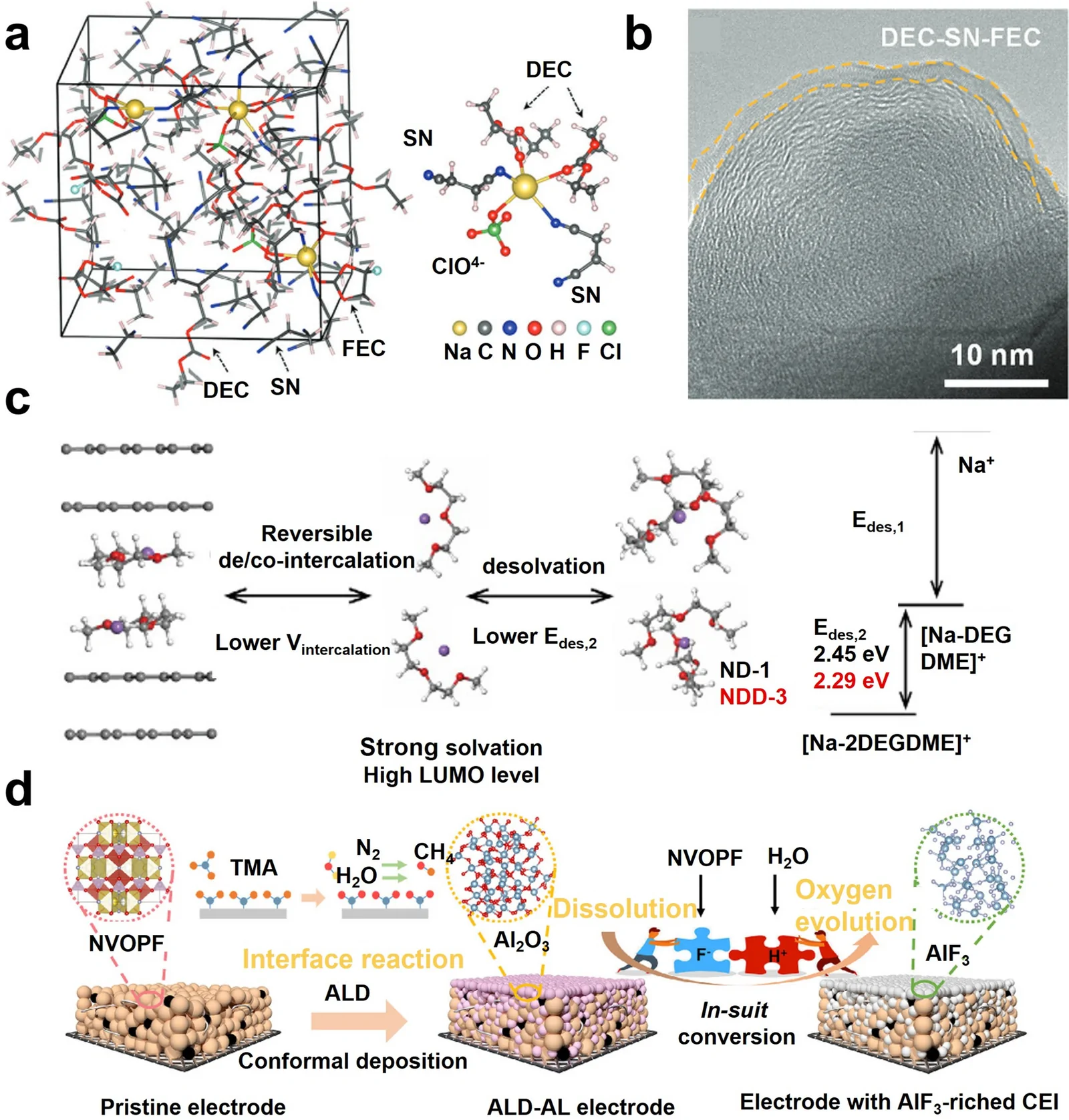
**a** Schematic illustration of Na^+^ ions-solvent co-intercalation into graphite [127]. Copyright 2021, Wiley. **b** HR-TEM images showing the CEI layer formed in electrolytes [124]. Copyright 2024, Wiley. **c** A snapshot of the DEC-SN-FEC electrolyte molecular system and a representative Na^+^ ions solvation structure [124]. Copyright 2024, Wiley. **d** Schematic illustration of the in-situ conversion process from ALD-AL to electrode with AlF3-rich CEI [123]. Copyright 2025, Wiley

The electrolyte is pivotal for resolving high-voltage interface stability issues. The composition of the electrolyte is a key factor for the electrolyte. By choosing an electrolyte with a special composition, a protective CEI film can be formed. At the molecular level, the preferential decomposition of electrolyte components is governed by the frontier molecular orbital energies and coordination states within the solvation sheath. For instance, in ionic liquid systems such as Na[FSA]-[C_3_C_1_pyrr][FSA], the decomposition pathway involves electron transfer from the cathode surface to the lowest unoccupied molecular orbital (LUMO) of the FSA^−^ anion and [C_3_C_1_pyrr]^+^ cation [126]. This triggers S–N and C–N bond cleavages, generating reactive radical intermediates that polymerize or react with Na^+^ to form a NaF-rich inorganic matrix embedded with sulfur/nitrogen species. The density and uniformity of the CEI layer arise from the balanced co-decomposition of both anion and cation, which prevents localized overgrowth and ensures complete surface passivation. In contrast, conventional electrolyte systems rely heavily on solvation structure modulation to dictate interfacial chemistry. The highly concentrated ether-based electrolyte (3.04 M NaPF_6_/DEGDME/DOL, NDD-3) exemplifies this principle [127]. Here, all DEGDME and DOL molecules are fully coordinated with Na^+^ ions, forming a contact ion pair (CIP) or aggregate (AGG)-rich solvation structure. This configuration elevates the HOMO energy level of coordinated solvents, making them less susceptible to oxidative decomposition (Fig. 14a). Instead, PF_6_^−^ anions within the primary solvation sheath are preferentially oxidized, decomposing to release F^−^ and forming a NaF-dominant CEI. The resulting CEI, approximately 5 nm thick (Fig. 14b), exhibits high interfacial energy and mechanical robustness, effectively suppressing solvent penetration and transition-metal dissolution. The role of specific functional groups in stabilizing the interface is further illustrated by the butanedione (SN)/DEC/FEC ternary system. SN, as the main solvent, contains nitrile groups (–C≡N) that strongly adsorb onto the NVOPF surface via lone-pair electron donation to surface V or Na sites, creating a solvent-derived priming layer (Fig. 14c) [124]. Upon oxidation, these nitrile groups undergo cyclotrimerization or react with Na^+^ to form NaₓN compounds, contributing to an inorganic-rich CEI. Concurrently, FEC additives undergo defluorination at high potentials, releasing HF that scavenges surface alkoxides and deposits NaF. The synergy between nitrile adsorption and FEC defluorination yields an ultrathin, insoluble CEI that blocks vanadium dissolution. This molecular-level interplay highlights the importance of designing electrolyte components with specific anchoring and decomposition functionalities.

The “double-C≡N group” additive strategy demonstrates how bifunctional additives can simultaneously modulate solvation structure and interfacial reactivity [128]. Succinonitrile (SN), containing two nitrile groups, exhibits strong chelation with Na^+^ ions, displacing solvent molecules from the primary solvation sheath and promoting anion-derived CEI formation. In-situ SFG and EC-SERS analyses reveal that SN adsorbs vertically on the cathode surface, forming a compact monolayer that directs subsequent decomposition of PF_6_^−^ ions. This molecular arrangement enhances oxidative stability and ensures uniform CEI deposition. Theoretical calculations confirm that the nitrile groups raise the reduction potential of Na^+^ ions -solvent complexes, thereby suppressing solvent co-intercalation and parasitic reactions. Moreover, the in-situ interfacial transformation strategy using an Al_2_O_3_ sacrificial layer underscores the importance of reactive fluorine capture at the molecular scale (Fig. 14d) [123]. The pre-deposited Al_2_O_3_ reacts spontaneously with F^−^ released from electrolyte decomposition, undergoing a conversion reaction to form AlF_3_. This process continuously anchors fluorine species, preventing their accumulation as HF and mitigating corrosion of the Al current collector. The resulting AlF_3_-rich CEI exhibits high ionic conductivity and chemical inertness, effectively stabilizing the cathode interface under high-voltage operation.

In summary, a molecular-level understanding of electrolyte design reveals that effective CEI formation hinges on three interconnected factors: (i) the preferential decomposition of anions or functional groups with high adsorption affinity, (ii) the reconstruction of solvation structures to suppress solvent reactivity and promote anion-derived CEI, and (iii) the incorporation of synergistic additives or sacrificial layers that modulate interfacial reactions in situ. Recent studies on solvation structures, such as high-concentration electrolytes, localized high-concentration designs, and functional group engineering, provide mechanistic insights that link electrolyte composition to CEI chemistry. Integrating these molecular-scale considerations into future electrolyte development will enable the rational design of high-voltage sodium-ion batteries with extended cycling stability and wide-temperature operability.

From a molecular-design perspective, the development of next-generation high-voltage electrolytes should prioritize solvents with intrinsically high oxidation resistance (e.g., sulfones and nitriles), sodium salts that favor inorganic interphase formation (such as FSA^−^- or PF_6_^−^-based salts), and functional additives that promote early-stage interfacial passivation (e.g., FEC). Concurrently, precise optimization of electrolyte concentration and component ratios is essential to minimize free solvent molecules and promote anion-dominated solvation structures, thus facilitating preferential anion decomposition. Finally, the construction of multicomponent electrolyte systems integrating primary solvents, co-solvents, and targeted additives offers a viable pathway to achieve long-term interfacial stability, high-rate capability, and extended cycle life in high-voltage sodium-ion batteries.

## Summary

As representative polyanionic cathode materials for sodium-ion batteries, NVPF and NVOPF have demonstrated considerable application potential owing to their robust three-dimensional NASICON frameworks, relatively high energy densities, and excellent thermal stability [129, 130]. In this review, we systematically compare the crystal structures and sodium storage mechanisms of NVPF and NVOPF, highlighting how anion chemistry and local coordination environments govern their electrochemical behavior. Furthermore, we summarize and contrast the synthesis strategies of these two materials, emphasizing that although a variety of preparation routes have been developed, achieving precise control over phase purity and morphology while simultaneously reducing process complexity and production cost remains a critical challenge for large-scale manufacturing.

To overcome the intrinsically low electronic conductivity of NVPF and NVOPF, a range of modification strategies such as carbon coating, nanostructure engineering, and elemental doping have been widely explored. These approaches have proven effective in enhancing electronic/ionic transport, alleviating kinetic limitations, and significantly improving rate capability and cycling stability. Beyond bulk properties, this review provides the first comprehensive summary of the failure mechanisms and high-voltage stability issues of NVPF- and NVOPF-based cathodes. While the degradation behavior of NVPF under high-voltage operation has been relatively well documented, the failure mechanisms of NVOPF remain far from fully understood, particularly with respect to interfacial reactions and long-term structural evolution, highlighting an important direction for future investigation.

The design principles derived from this comparative study, including anion-coordination regulation, Na^+^ ions/vacancy ordering modulation, and CEI engineering, are broadly applicable to other NASICON-type sodium cathodes, though their transferability is influenced by the specific electronic structure of the transition metal and the compatibility of synthesis routes. Looking forward, several critical research directions warrant focused attention to accelerate the practical deployment of NVPF- and NVOPF-based cathodes. First, synthesis process innovation should prioritize scalable, cost-effective, and environmentally benign routes. The room-temperature mechanochemical solid-state method has demonstrated kilogram-scale feasibility for NVOPF, yet challenges in morphological uniformity and fluorine retention persist. Developing continuous processing strategies and establishing pilot-scale production platforms are crucial steps toward industrial viability. Second, deepening the understanding of anion chemistry at the atomic level is essential. While O^2−^/F^−^ ions substitution has been shown to modulate redox potentials and diffusion kinetics, the precise roles of other anionic species (e.g., Cl^−^, Br^−^, SiO_4_^4−^, SO_4_^2−^) in tailoring local coordination environments, electronic structures, and phase transition pathways remain underexplored and warrant systematic investigation through combined theoretical calculations and advanced characterization techniques. Third, interfacial stability engineering requires a shift from empirical electrolyte formulation to mechanistic design principles. The composition and structure of the CEI critically determine high-voltage cycling stability; therefore, future efforts should focus on elucidating the dynamic evolution of CEI under realistic operating conditions and designing electrolyte systems that promote the formation of thin, inorganic-rich, and mechanically robust interphases. Fourth, full-cell evaluation under practical conditions must become a standard practice. Most existing studies report half-cell performance under idealized conditions, whereas compatibility with hard carbon anodes, electrolyte starvation effects, electrode loading, and long-term cycling stability at elevated temperatures are key metrics that dictate real-world applicability. Addressing these interconnected challenges through interdisciplinary collaboration will be essential to translate the intrinsic advantages of NVPF and NVOPF into commercially viable sodium-ion battery technologies.
